# Nanoprobe synchrotron X-ray fluorescence microscopy reveals selenium-rich spherical structure in mouse retinal pigment epithelium

**DOI:** 10.1038/s41598-025-11678-4

**Published:** 2025-08-01

**Authors:** Marta Ugarte, Kalotina Geraki, Elizabeth Bentley, Roger Cox

**Affiliations:** 1 Faculty of Biology, Medicine and Health, University of Manchester, Oxford Road, Manchester, M13 9PL UK; 2 Manchester Royal Eye Hospital, Manchester University NHS Foundation Trust, Oxford Road, Manchester, M13 9WL UK; 3 Diamond Light Source, Harwell Science and Innovation Campus, Didcot, Oxfordshire, OX11 0DE UK; 4 MRC Harwell Institute, Mammalian Genetics Unit, Harwell Campus, Oxfordshire, OX11 0RD UK

**Keywords:** Outer retinal complex, Iron, Zinc, Copper, Selenium, Nanobeam X-ray fluorescence microscopy, Photoreceptor outer segment phagocytosis, Synchrotron, Cell biology, Diseases, Medical research

## Abstract

High-resolution nano-focus X-ray fluorescence microscopy using hard X-rays at the European Synchrotron Radiation Facility (ESRF) IDB16 beamline detected endogenous barium, bromine, calcium, chlorine, copper, iron, manganese, potassium, phosphorus, rubidium, sulphur, selenium, strontium and zinc, at tissue, cellular and subcellular level in the outer retinal complex of light adapted, 3-week-old, male C57BL6 mice. Fresh snap-frozen (20 μm) cryosections dried at room temperature were scanned at 1 μm, 300 nm and 50 nm spatial resolution by incident X-ray photons from the synchrotron beam. Analysis of 2D maps and 3D surface plots by PyMCA and ImageJ revealed elevated zinc concentrations in the choriocapillaris (CC) (mean 45, range 28–77 ppm), retinal pigment epithelium (RPE) layer (mean 47, range 20–76 ppm), photoreceptor inner segments (RIS) ellipsoid zone, outer limiting membrane (OLM) (mean 32, range < 1–44 ppm) and outer nuclear layer (ONL) in between photoreceptor cell bodies. Mūller cells processes in ONL and their interdigitations in RIS ellipsoid zone seem to contain zinc in the cell membrane. Iron was found at elevated amounts in RIS myoid zone (mean 38, range 14–68 ppm), RPE layer (52, range 24–143 ppm), and choroid (60, range 36–172 ppm). Copper was also detected in the CC (4.3, range 1.9–9.7 ppm), RPE layer (4.5, range 1.6–20.8 ppm), and RIS myoid zone (4.9, range 1.25–10.2 ppm). Calcium was found with granular/punctate distribution in OLM (159, range 49–962 ppm), RIS myoid zone (245, range 36-1370 ppm), RPE layer (1134, range 257–2503 ppm), and CC (1101, range 323–2090 ppm). The metalloid selenium was present in the CC (1.8, range < 1-4.7 ppm] and across the RPE (basal, central, apical) (2.4, range < 1-8.5 ppm). High resolution maps of the interface photoreceptor outer segments (ROS) and the RPE apical side revealed selenium-rich spherical structures (appr. 1 μm diameter) (mean 5.6, range 2.2–8.1 ppm), associated with calcium (mean 1057, range 619–1755 ppm), phosphorus (9924, range 6118–15058 ppm), and manganese (0.7, < 1–24 ppm), surrounded by a zinc-containing layer. This study presents the first nanoprobe X-ray fluorescence microscopy image analysis of adult mouse light adapted outer retinal complex from the whole tissue to subcellular structures. The high spatial resolution (location) and high sensitivity (metal quantity) findings, together with the information on biometals available in the literature, allowed us to propose a schematic model of possible selenium biological processes and their role in physiological activities in the outer retinal complex. We hypothesise there is a dedicated selenium-rich spherical structure with the ability to cross RPE cell membranes (i.e. the outer blood retinal barrier) and with potential roles in certain biological function(s) (e.g. ROS phagocytosis by RPE cell microvilli, trans-RPE transport).

**Supplementary Information:**

The online version contains supplementary material available at 10.1038/s41598-025-11678-4.Numbers of maps and surface plots for all maps need revision. I cannot amend pdf.

## Introduction

Metal(loid)s play pivotal roles in retinal and choroidal physiology and disease^1–3^. However, not much is known about their cellular and subcellular distribution, concentration or associations. We set out to answer the scientific question: what is the metal and metalloid composition of the adult mouse light-adapted outer retinal complex, at tissue, cellular and subcellular level?

**Fig. 1:**
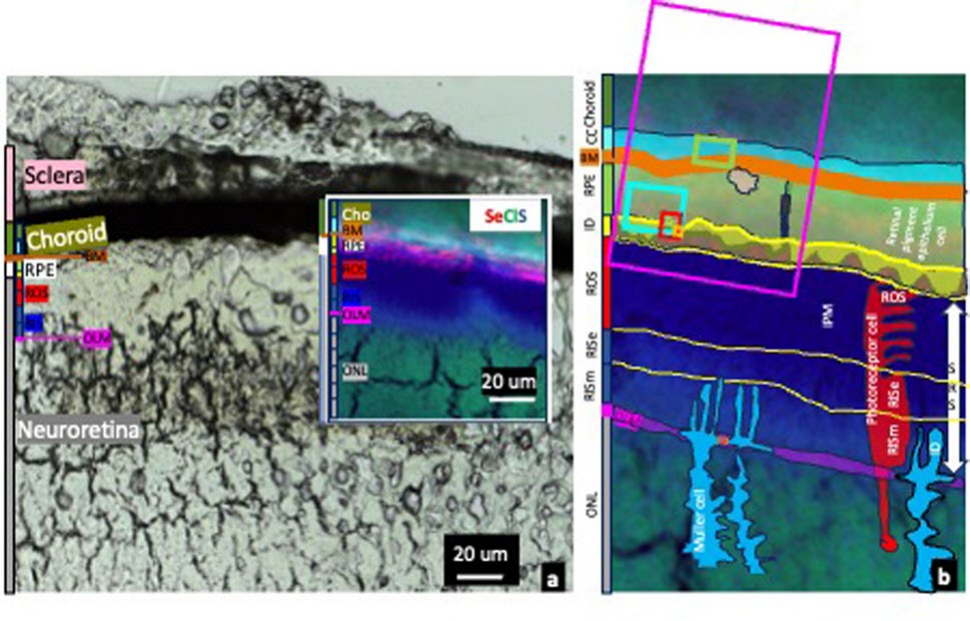
Light transmission photograph and overlay X-ray fluorescence microscopy trace elements map (red, selenium; green, chlorine; blue, sulphur) **(a)**, together with schematic representation showing the different layers and cellular structures in the outer retinal complex **(b)**. The outer retinal complex includes neuroretina (grey bar), retina pigment epithelium (RPE) (white bar) and choroid (green bar). It contains various compartments (i.e. intra and extracellular compartments, cell membranes, The outer nuclear layer (ONL) contains cell bodies of photoreceptors (i.e. neurones), Muller cells (glial cells) and extracellular matrix. The outer limiting membrane (OLM) consists of Müller cell-to-Müller cell and Müller cell-to-photoreceptor cell projections with adherens junctions (pink bar). The photoreceptor inner segments myoid region (RISm) consists of apical microvilli extensions of Müller cells (interdigitations) and inner segments of photoreceptor cells. The RIS ellipsoid (RISe) region contains photoreceptors inner segments with mitochondria and endoplasmic reticulum. The photoreceptor outer segments (ROS) layer, the site of phototransduction, includes OS and the interphotoreceptor matrix (IPM). The RPE layer, the outer part of the blood retinal barrier, is formed of a polarised monolayer with tight junctions (black bar) with distinct ultrastructural features and specialized functions in the apical and basolateral part. The apical part has numerous microvilli interdigitating with and ensheathing the tips of the ROS (RPE interdigitations-ID layer, yellow bar). The RPE basal side is marked by plasma membrane infoldings resting on the basement membrane, Bruch´s membrane (BM). The “subretinal space” (SRS) is the region between the RPE apical processes and the OLM, containing ROS, IPM and RIS (i.e. both ellipsoid and myoid regions). Inset in (b) mark areas included in Map 2 (pink box) (Figs. 5 and 8), Map 3 (green box) (Figs. 5 and 8), Map 4 (pale blue box) (Figs. 5 and 8), Map 5 (red box) (Figs. 5, 6 and 8) and Map 6 (ocre box) (Figs. 5, 7 and 8).

The outer retinal complex is a very well organised structure with several layers and interfaces, and a heterogenous distribution of neurons (i.e. photoreceptors), glial cells (i.e. Müller cells, astrocytes, microglia), retina pigment epithelial (RPE) cells (the outer part of the blood retinal barrier, BRB), extracellular membranes [e.g. Bruch´s membrane (BM)], matrices [e.g. interphotoreceptor matrix (IPM)] and vascular tissue (i.e. choroid) (Fig. 1). The outer nuclear layer (ONL) contains photoreceptors cell bodies, Müller cells processes and extracellular matrix (ECM). The outer limiting membrane (OLM) consists of Müller cell-to-Müller cell and Müller cell-to-photoreceptor projections with cell adherens junctions; and provides structural support for cellular organization, integrity, and alignment^4^. The “subretinal space” (between the OLM and RPE), contains photoreceptors inner segments (RIS) (both ellipsoid and myoid regions/zones), ROS and IPM. The RIS myoid region contains the photoreceptor segments portion closer to the cell body and Müller cells apical microvilli extensions. The ellipsoid region includes the part of the RIS with mitochondria and endoplasmic reticulum. The RPE is a polarised monolayer with tight junctions and distinct ultrastructural features and functions in the apical and basolateral regions. The apical cell membrane has numerous microvilli (3–7 μm long) interdigitating with and ensheathing the ROS tips. The basal side is marked by plasma membrane infoldings resting on BM, which separates it from the underlying vascular tissue, the choroid.

The outer retina complex has multiple functions including phototransduction by photoreceptors; engulfment and phagocytosis of ROS, absorption of light energy, metabolic/biochemical functions and transport by the RPE cells (Table 1)^5–7^. It is the primary site of dysfunction in age-related macular degeneration (AMD), some inherited (e.g. ABCA4-related retinopathy) and autoimmune disorders (e.g. choriocapillaridopathies)^8–10^. Many of these diseases have no effective treatments to date. We are of the opinion that some of the keys to their clinical management may lie in the understanding of metal(loid)s biology in the outer retinal complex.

The electron configurations, valences and various oxidation states of biometals (e.g. iron, zinc, copper, manganese, calcium)^11–14^, and the metalloid, selenium^15,16^, allow them to react chemically and combine with other elements. They are integral components of nearly all biological processes. They are involved in electron transfer processes, intra and intercellular communication, maintenance of electrical charges, osmotic pressure, as well as the structure and function of nucleic acids^17,18^ and more than 40% of all enzymes^19,20^. They act as: (1) co-factors, (2) catalysts, (3) regulators (e.g. adenosine triphosphate, ATP, hydrolysis), and/or (4) structural components (e.g. zinc fingers).

Specific functions of the outer retinal complex, which are likely to depend heavily on the contribution of biometal(loid)s include: (1) phototransduction, (2) retinal dark current, (3) ROS phagocytosis by the RPE, (4) trans-RPE transport (i.e. transmembrane, vesicle-mediated, along microtubules, endocytosis/exocytosis), (5) visual cycle and (6) retinal adhesion. These functions require a tightly controlled microenvironment with high amounts of substrates, energy and antioxidant defence, together with rapid modifications of transmembrane transport^21,22^.

Various methods are currently available to evaluate the distribution and concentration of elements in biological tissue [i.e. synchrotron X-ray fluorescence microscopy, scanning electron microscopy coupled with energy dispersive X-ray spectroscopy (EDXRS), secondary ion mass spectrometry (SIMS), laser ablation inductively coupled plasma mass spectrometry (LA-ICP-MS), particle induced X-ray emission (PIXE), confocal microscopy with fluorophores, autoradiography with radioactive isotopes]^23–28^. Among them, the analytical technique synchrotron X-ray fluorescence microscopy offers extraordinary capabilities. It can simultaneously detect various elements with high spatial resolution, high sensitivity and relatively fast scanning time^29,30^. It is non-destructive, and the samples can, in most cases, be examined at room temperature, under normal atmospheric conditions without chemical preparation. Synchrotron X-ray fluorescence microscopy uses incident X-ray photons, from the synchrotron beam, to excite the inner shell electrons of elemental atoms in the tissue sample. When the system relaxes, electrons transition from higher energy levels to the vacant inner shell and release X-ray fluorescence photons of characteristic energies. Energy dispersive detectors coupled to electronics, record them allowing fingerprinting of the element they originate from. The range of elements detected depends on the excitation energy used and the beamline capabilities.

We used the synchrotron beamline ID16B^31^ at the ESRF. which is best suited for investigating elemental composition at the subcellular scale, by using nanoprobe X-ray fluorescence microscopy. The current characteristics of this beamline include: (1) the use of hard X-rays, (2) low detection limits of light (e.g. phosphorus), as well as heavy (e.g. bromine) elements, (3) excellent spatial resolution (as low as 50 nm) and (4) fast detection of emitted fluorescence resulting at short scanning times.

Our study will set baseline information for future studies to elucidate the role and homeostasis of metal(lloid)s in retinal function and disease, and the development of potential new therapies.

**Table 1:** Summary of the components and functions of different layers in the outer retinal complex. BM, bruch´s membrane; ECM, extracellular matrix; IPM, interphotoreceptor matrix.

	LAYER						
	Inner choroid	Choriocapillaris CC/BM	Retinal pigment epithelium RPE	Photoreceptor outer segments ROS	Photoreceptor inner segments RIS	Outer limiting membrane OLM	Outer nuclear layer ONL
**CELLULAR** **EXTRA-CELLULAR**	Melanocytes Stromal cells Blood cells Endothelial cells Smooth muscle cells ECM Interstitial fluid	Melanocytes Stromal cells Blood cells Endothelial cells Smooth muscle cells Glial cells ECM/BM Interstitial fluid	RPE basal central apical parts	ROS IPM	r RIS ellipsoid zone s RIS myoid zone Muller cell interdigitations ECM	Mūller cells-to-Muller cells processes Muller cell-to-photoreceptor processes	Photoreceptors cell bodies Muller cell processes ECM
**FUNCTION**	Nutrition support Thermal support Light absorption	Transport of nutrients, ions and water Thermal support Light absorption	ROS phagocytosis Transport of nutrients, ions and water Secretion of factors for retinal integrity Light absorption Photooxidation protection Visual cycle	Phototransduction	Metabolic Biochemical	Structural support Barrier	Metabolic Biochemical

## Methods

### Animals

Mice (*n* = 3) were maintained following UK Home Office legislation and local ethical guidelines issued by the Medical Research Council (Responsibility in the Use of Animals for Medical Research, July 1993). Procedures were approved by the MRC Harwell Animal Welfare and Ethical Review Board (AWERB). All procedures adhered to the tenets of the Declaration of Helsinki^32^. Mice were maintained in an animal room with 12 h light and dark cycle with a temperature of 21 ± 2 °C and 55 ± 10% humidity. Mice were fed *ad libitum* a standard rat and mouse No. 3 breeding diet (RM3; Special Diets Services, France).

### Sample preparation

Tissue was collected in the morning, a few hours after the lights in the animal house went on. Retinae, therefore, were light-adapted. Immediately after humane sacrifice by neck dislocation, one eye from each of the 3-week-old C57BL/6 mice (*n* = 3) were snap-frozen in liquid nitrogen at −196^0^ C (−320^0^ F) (this is likely to result in rapid cessation of metabolic activity). Whole-eye 20-µm thick sections were cut with a cryostat, placed on silicon nitride membranes (trace-element-free support mounting material, thinner than tissue sections)^33^ and allowed to air-dry. Stainless-steel cryostat blades were regularly inspected and changed to ensure the absence of defects, oils, or rust. The RPE and photoreceptor cells have an approximate average 10 μm thickness^34^. A 20-µm-section is expected to have 1 layer of intact cells in the centre with the cytosol retained as it dries. Once cut, the thickness and topography of the sections were checked under the microscope to avoid thickness artefacts at the time of X-ray fluorescence scanning (e.g. increased scatter and higher apparent fluorescence counts)^35^. Air-dried sections were scanned within 7 days from cutting.

The tissue samples should represent their native state in the living animal, as much as possible, while at the same time being sustainable for X-ray fluorescence analysis with detectable fluorescence signal of multiple elements. We aimed to preserve the morphology and integrity of cellular and subcellular structures and prevent loss and/or redistribution of chemical elements. Potassium and chlorine (which exist in tissues as ions, K^+^ and Cl^−^) can potentially move with physical (drying, dehydration) or chemical (infiltration of resins) stabilization^36,37^. Phosphorus, sulphur and selenium (which may be volatile) can be lost over time after cutting^29^.

### Synchrotron X-ray fluorescence (XRF) microscopy

**Fig. 2:**
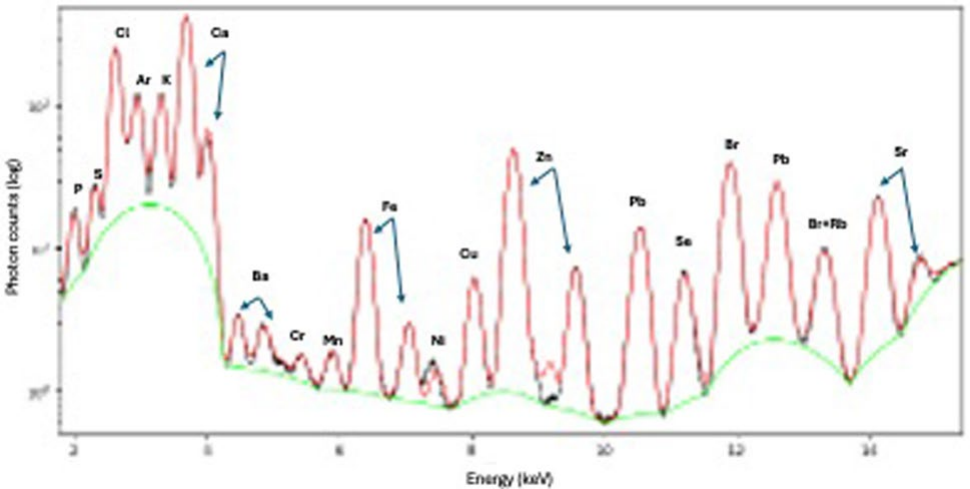
Example of a spectrum and elemental composition at the point where the synchrotron X-ray bean hit the sample. The number of photon counts (logarithmic scale) (y axis) is plotted versus the photons characteristic X-ray fluorescence energy (x axis), with peaks associated with specific elements of the sample. The black line is the raw data, green line is the background used for the fit, and red line is the fitted data (NB, lead signal originates from beamline background).

We used beamline ID16B at the ESRF {31] with the excitation energy set to 17.5 keV. The X-ray beam was focused by Kirkpatrick–Baez mirrors to 50 nm. The photons emitted from the sample were detected by two silicon drift energy dispersive detectors that offer efficient detection of light and heavy elements (phosphorus to bromine in this case). The scanning mode was continuous with 100–400 msec dwell time. An energy spectrum was acquired from each point in the maps. An example is shown in Fig. 2. The number of photons is plotted against the photon energy. The sharp peaks originate from the specific atomic excitations and the elemental composition.

Three spatial resolution levels were used: 1 μm, 300 nm and 50 nm. The highest (50 nm) was achieved when the sample was at the optimum focal distance from the focusing mirrors and mapping steps were 50 nm. For the intermediate scans, the sample remained in the focal plane but the mapping step was increased to 300 nm. For the 1 $\:\mu\:$m, the sample stage was moved “off the focal plane” for broader projected beam size and 1 $\:\mu\:$m steps used. The latter allowed us relatively quick survey of the samples to determine tissue landmarks, areas of enriched elemental concentrations and regions of interest (ROI) for subsequent analysis at higher resolution (300 nm and 50 nm). Table 2 shows the details of the six maps acquired from one of our animals and shown here.

**Table 2:** Details of X-ray fluorescence microscopy maps (Map 1–6), together with the outer retinal complex layers included in each map: BM, bruch´s membrane. CC, choriocapillaris. Chor, choroid. ID, interdigitations. ONL, outer nuclear layer. RISe, photoreceptor inner segments ellipsoid zone. RISm, photoreceptor inner segments myoid zone. ROS, photoreceptor outer segments. RPE, retinal pigment epithelium.

		Area µm x µm	Dwell time msec	Resolution nm	Step nm	Retina and choroidal layers included
Map 1	Figure 4 Figure 5	95 × 94	400	300	300	Cho, CC, BM, RPE, RPE ID, ROS, RISe, RISm, OLM, ONL
Map 2	Figure 5	25 × 37	400	300	300	CC, BM, RPE, RPE ID, ROS
Map 3	Figure 5	6 × 4	400	50	50	CC, BM, RPE basal
Map 4	Figure 5	9 × 7	400	50	50	RPE apical, RPE ID
Map 5	Figure 6	4 × 3	400	50	50	RPE ID
Map 6	Figure 7	1.8 × 1.8	400	50	50	RPE ID/ROS interface “vesicle”

Cryofixed air dried tissue sections were scanned at room temperature under normal atmospheric pressure. The stability of the samples under our experimental ambient conditions is anticipated to have been well maintained^38^.

Cryogenic sample environment, under frozen hydrated conditions, is the preferred choice for single cell, homogenous cell populations and one-cell-layer tissues analysis^39,40^. However, cryogenic analysis of composite tissue sections, like the outer retina complex, can be associated with artefacts^41^, such as spatial resolution degradation and loss of X ray photons emitted from light elements (e.g. phosphorous and sulphur), which can be absorbed by water. The outer retinal complex has diverse cell types. Its layers have non-homogenous distribution of water, free elements and hydrophilic proteins. Scanning of hydrated samples might result in non-homogenous redistribution.

### Outer retinal complex metal concentration analysis

**Fig. 3:**
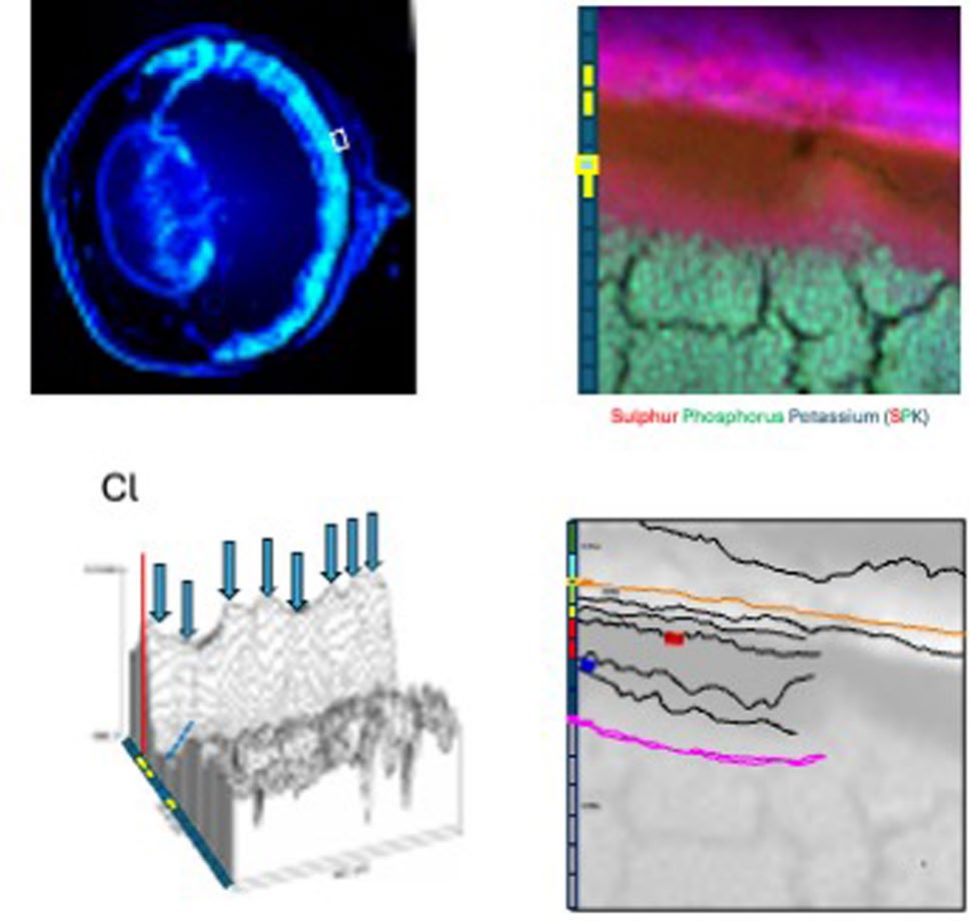
**(a)** Three-week old male C57BL6 mouse whole eye section and localisation (white box) of outer retinal complex analysed by synchrotron X ray fluorescence microscopy (Map 1) **(b)**. DAPI staining was used for nucleus identification. The area scanned was within 1 mm from the optic nerve. We used ImageJ 2D colour maps (b) and 3D surface plots of chlorine **(c)**, phosphorus and sulphur to delineate tissue landmarks and interfaces. The 3-D plots highlight the spatial relationship among elements and regional differences. The z-axis represents the pixel concentration (ppm). based on the detection of the pixels of maximum intensity along the *z*-axis with a steep slope at the edge, we identified putative pixels likely to belong to a biological interface (blue arrows in 3-D-plots). We, then, connected adjacent putative pixels satisfying natural proximity constraints. We, subsequently extracted individual layers in the outer retinal complex manually **(d)**.

The areas scanned were within 1 mm from the optic nerve (Fig. 3). The X-ray fluorescence photons emitted by 14 elements (barium, bromine, calcium, chlorine, copper, iron, manganese, potassium, phosphorus, rubidium, sulphur, selenium, strontium, zinc) were measured simultaneously at every pixel. At the time of the experiments, the ESRF did not have a “continuous top-up” mode of operation for injection of electrons to keep the stored electron beam current relatively constant^42^. There was a 12 h cycle refill. All our maps were, therefore, rigorously normalized to the corresponding incident X-ray flux. Two reference materials, AXO thin film XRF (RF-200-S2371; AXO Dresden GmbH) and NIST SRM 1577 C (bovine liver powder pressed into a self-supporting pellet)^43,44^ were measured to facilitate the calculation of elements concentration levels in the samples.

PyMca^45^ was used for peak fitting of the X-ray spectra (Fig. 2) and for quantification. PyMca deconvolves contributions of neighbouring peaks and the background is removed. Quantification was achieved by modelling the matrix of the reference materials and the samples, allowing the conversion of fluorescence peak intensities to concentrations in ppm. The effects of geometry, signal absorption by the sample, atmosphere and detectors were all taken account of. Concentration maps were then converted into tiffs for statistical processing in ImageJ software^46^.

### Delineation of outer retina landmarks, interfaces, layers and ROI

We used ImageJ 2D colour maps and 3D surface plots of chlorine (the component of chloride, Cl^−^, main extracellular ion), potassium (intracellular ion), phosphorus (bound to phospholipids in cell membranes and phosphate groups in DNA and RNA) and sulphur (component of proteins with methionine and cysteine aminoacid residues), to delineate tissue landmarks and interfaces (Fig. 3). The 3-D plots highlight the spatial relationship among elements and regional differences in the context of tissue compartments, cell diversity and layers organisation. The x and y axes in the 3D plots represent the distance along the horizontal and vertical axes of 2D maps. The z-axis represents the pixel concentration (ppm).

We extracted individual layers in the outer retinal complex manually, as shown in Fig. 3 and Supplementary material. This allows very accurate localization. However, it is a very laborious process, which requires the need to know beforehand the structure and relative positions of interfaces. Based on the detection of the pixels of maximum intensity along the *z*-axis with a steep slope at the edge, we first identified putative pixels likely to belong to a biological interface (blue arrows in 3-D-plots). We, then, connected adjacent putative pixels satisfying natural proximity constraints.

ROI (i.e. tissue layers) were subsequently set in ImageJ for further analysis and concentrations of the 14 elements extracted. Mean, minimum and maximum (range) values were calculated. Threshold analysis was used in certain cases to facilitate extraction of concentrations from specific features and structures.

## Results

**Fig. 4:**
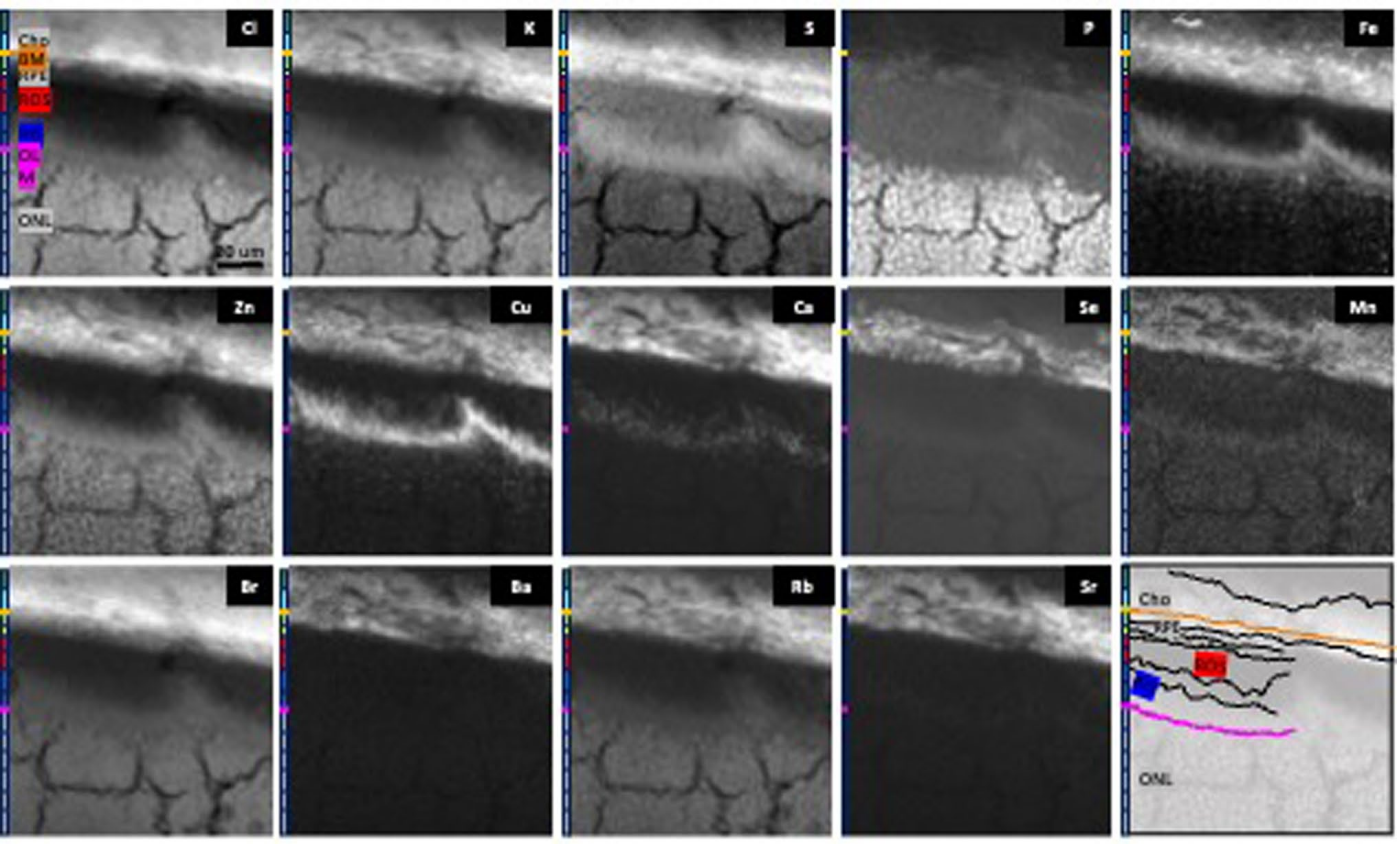
Example of 2-D maps X-ray fluorescence microscopy of 3-week-old male C57BL6 mouse outer retinal complex (Map 1, 94 × 95 µm^2^). The maps include outer nuclear layer (ONL), outer limiting membrane (OLM), photoreceptor inner segments (RIS) (ellipsoid and myoid regions), photoreceptor outer segments (ROS), retinal pigment epithelium (RPE) (interdigitations, apical zone, central zone, basal zone), Bruch´s membrane (BM), choriocapillaris (pale blue bar) and inner choroid (Cho). The 2D colour maps and corresponding 3-D surface plots (see Supplementary material) of chlorine (Cl), potassium (K), sulphur (S), phosphorus (P) allowed accurate delineation of tissue landmarks, layers and interfaces, as highlighted in (o). Some layers showed elevated concentrations of specific elements.

**Fig. 5:**
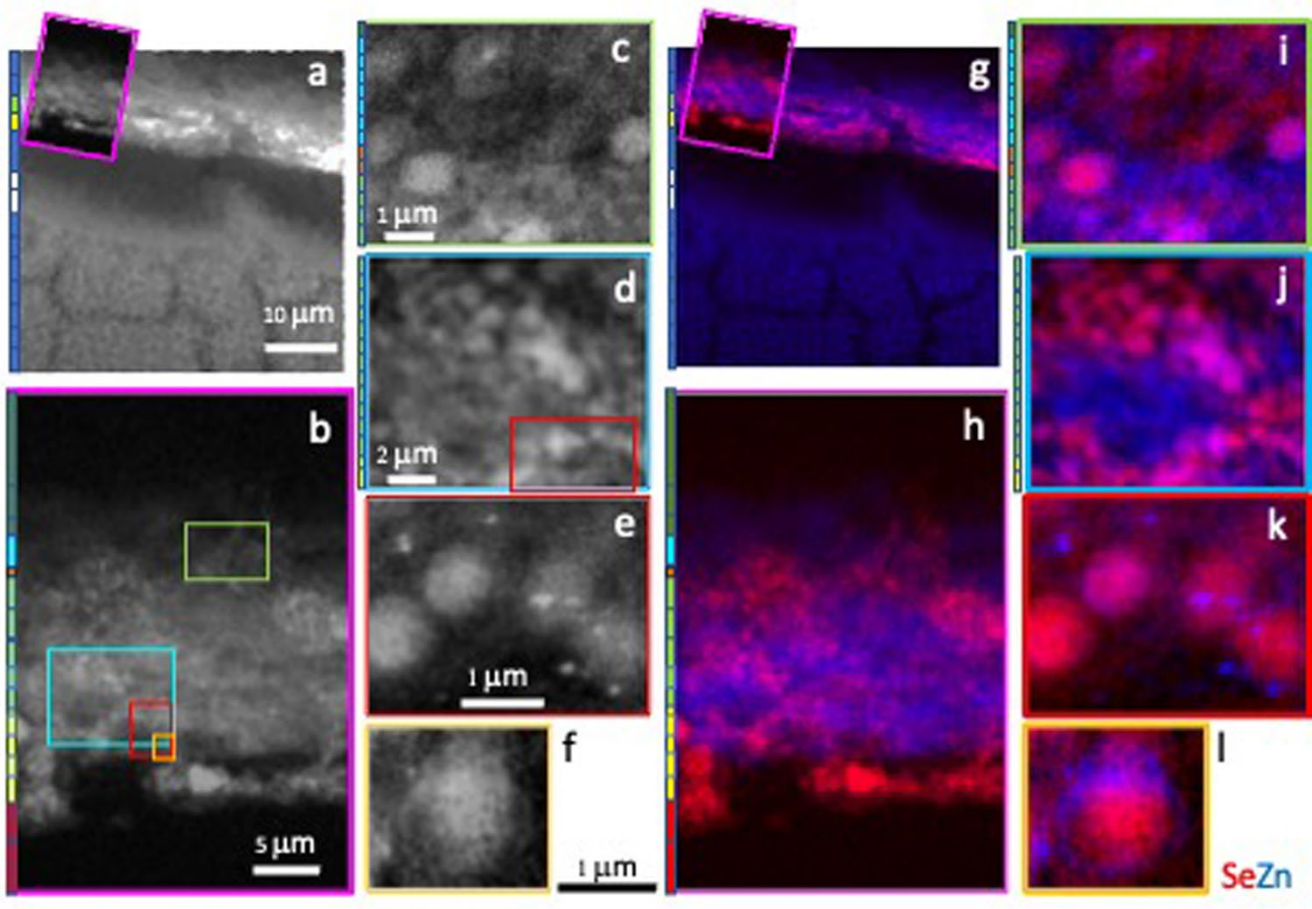
Merged 2D maps selenium (Se) (red) and zinc (Zn) (blue) **(a**,** b**,** c**,** d**,** e**,** f)** of differing spatial resolution of adult male C57BL6 mouse light adapted outer retinal complex. Inset boxes (pink, green, pale blue, red, ocre) mark the areas scanned in each map. Black and white images (g, h, I, j, k, l) are included to help identify the areas shown in all the maps. Map 1 **(a)** (as in Figs. 1, 4 and 5a and g). Pink box inset marks the area of Map 2 in **(b)** (25 × 37 µm^2^). **(c)** Map 3 (6 × 4 µm^2^) (green box). **(d)** Map 4 (9 × 7 µm^2^) (pale blue box). **(e)** Map 5 (3 × 4 µm^2^) (red box). **(f)** Map 6 (1.8 × 1.7 µm^2^) (ocre box).

**Fig. 6:**
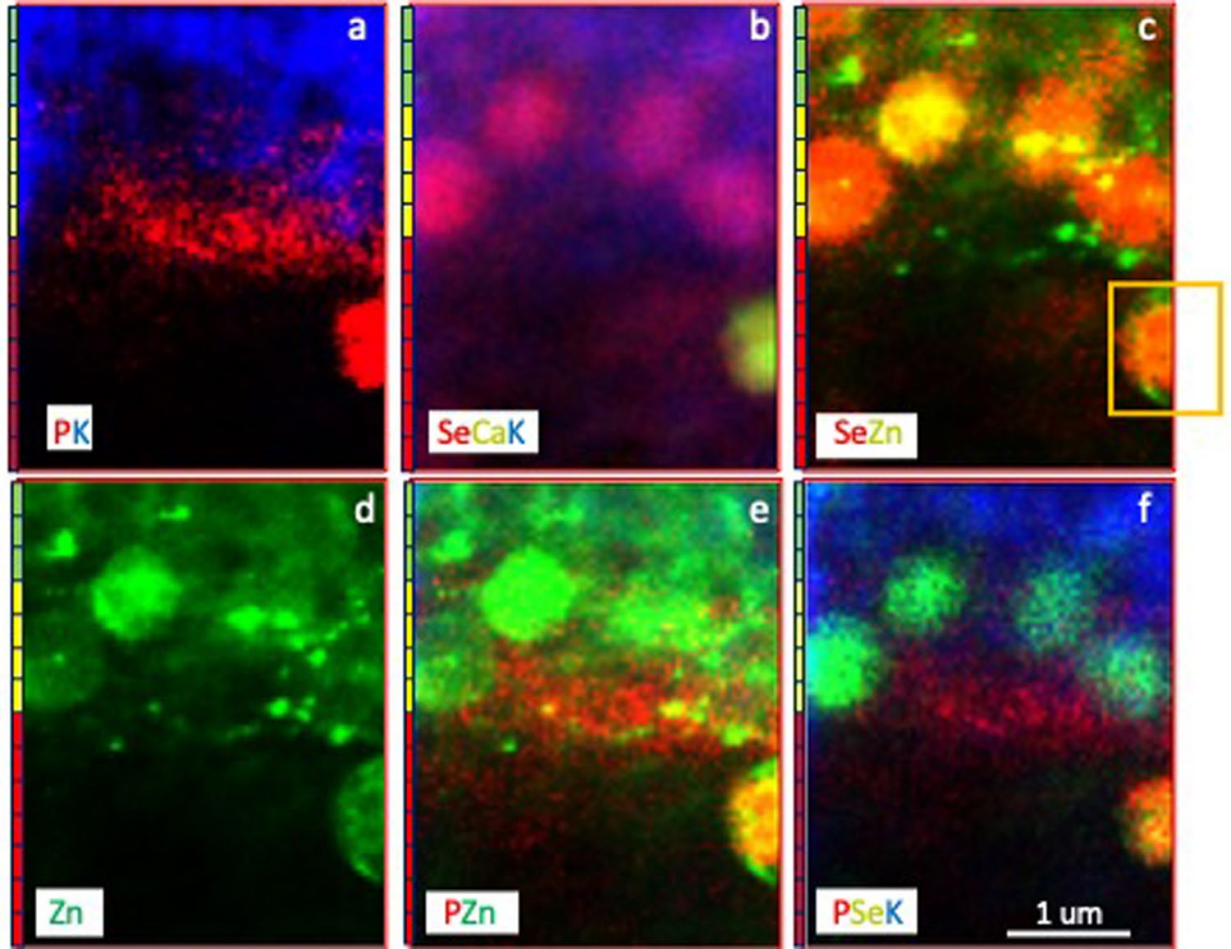
2-D multielement X-ray fluorescence microscopy images of Map 5 (3 × 4 µm^2^) (inset, red box marked in Fig. 5b, d and j). Map 5 includes apical RPE (green bar), RPE interdigitations (ID) (yellow bar) and ROS tips (red bar). From left to right, 4 selenium rich spherical structures are inside the RPE (using potassium as a reference for intracellular environment). The 5th vesicle (half scanned) (ocre box) seems to be extracellularly at the interface RPE interdigitations/ROS tip.

**Fig. 7:**
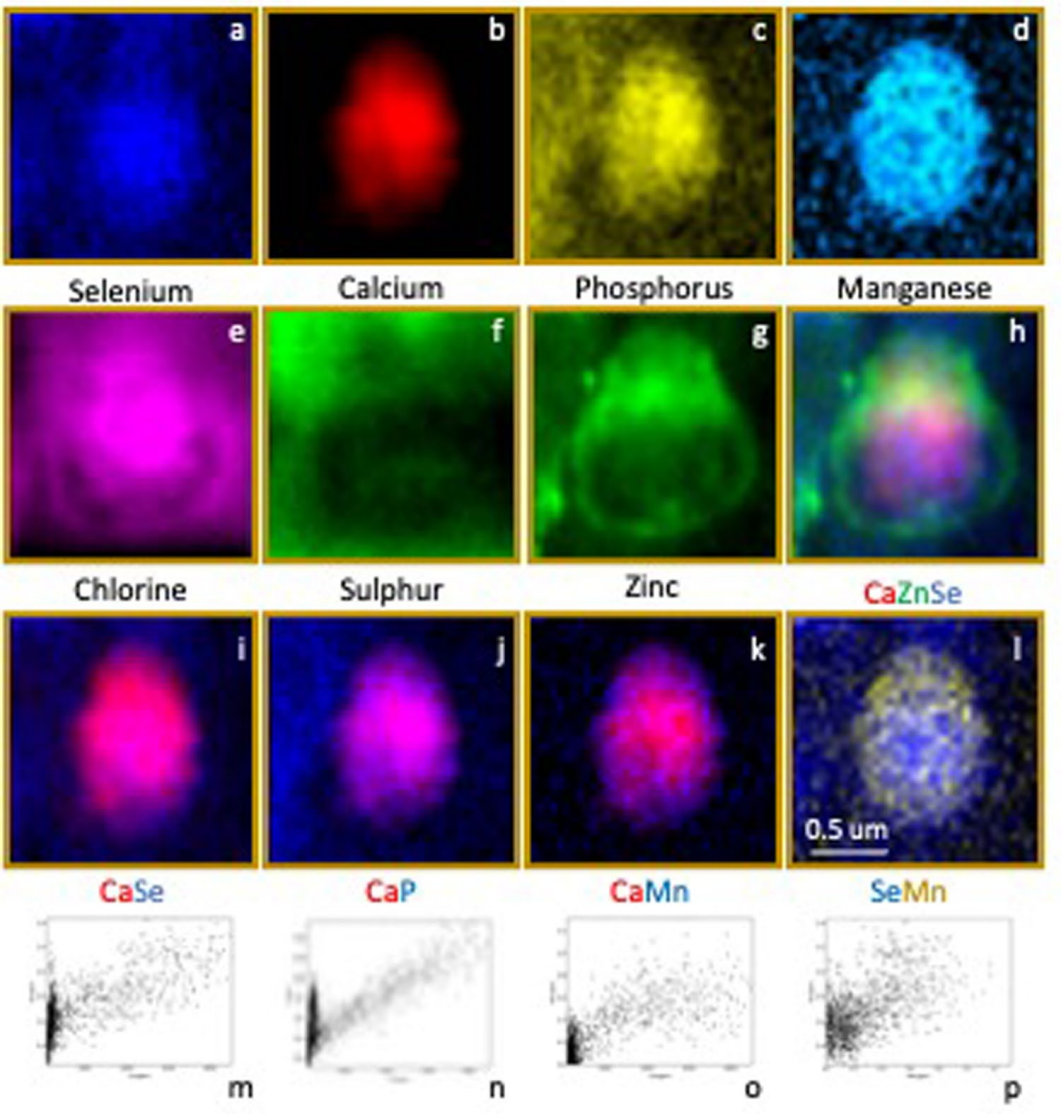
Multielement 2-D colour maps of Map 6 (1.8 × 1.7 µm^2^) (a-l) and correlations of calcium and selenium (m), calcium and phosphorus (n), calcium and manganese (o) and selenium and manganese (p) of single selenium-rich spherical structure at the interface between the RPE interdigitation/photoreceptor tip of ROS.

We obtained six 2D maps (Map 1–6), and the corresponding 3D surface plots, with intermediate (300 nm) and high (50 nm) spatial resolutions (Table 2), containing different layers and cellular regions, for 14 elements (barium, bromine, calcium, chlorine, copper, iron, manganese, potassium, phosphorus, rubidium, sulphur, selenium, strontium, zinc). Maps 1 and 2 at 300 nm resolution. Maps 3–6 at 50 nm. Insets in Map 1 (Fig. 5a, Fig. 5g) and Map 2 (Fig. 5b) mark the higher resolution scans. Pink box, Map 2; pale blue box, Map 3; green box, Map 4; red box, Map 5; ochre box, Map 6. Map 1 (Figs. 4 and 5a and g) contains part of the photoreceptors in the neuroretina, RPE and choroid. Map 2 (Fig. 5b and h) includes the RPE together with the surrounding tissues on the basal and apical sides, the BM/CC/inner choroid and top of ROS, respectively. Map 3 (Fig. 5c and i) shows a section of basal RPE, BM/CC. Map 4 (Fig. 5 d and j), in turn, shows an intracellular portion of RPE cell. Map 5 (Figs. 5e and k and 6) includes RPE apical microvilli and ROS tips. Map 6 (Fig. 5f and l Fig. 7) depicts a vesicle-like structure, at the interface between RPE microvilli and top of ROS.

Chlorine was found in high amounts in the choroid (mean 5989 ppm, range 3950–8735) with a sharp drop in concentration at the interface between the innermost part of the choroid (i.e. CC) and BM/RPE basal membrane. Chlorine concentration in the RPE was 4023 ppm (range 2072–7145) (Table 3). Potassium was found in high amounts within the RPE (mean 6169 ppm, range 2534–10740) and cell bodies of photoreceptors (mean 4874 ppm, range 521–6775) (and possibly Müller cells) in the ONL. Phosphorus was found in high amounts in photoreceptor cell bodies in the ONL (mean 21074 ppm, range 1160–41903), RIS (mean 9503 ppm, range 2983–18338) and ROS (particularly their tips) (mean 8541 ppm, range 1953–16270). Sulphur was found at the highest concentration in layers corresponding to CC (mean 7177 ppm, range 3359–10450) and RPE (mean 7601 ppm, range 4745–11067). The combination of chlorine, potassium, phosphorus and sulphur maps allowed us to delineate tissue landmarks, interfaces and layers (Fig. 4).

Iron was detected, in the form of “hotspots”, in the choroid, RPE, RIS myoid region and ONL. A cluster of 1 μm long rod-like structures was found intracellularly in RPE cell apical part “cladding” a selenium-containing structure. (Supplementary material). Zinc was predominantly localised to the choroid, RPE cell body and interdigitations, RIS myoid zone, OLM and ONL. In the ONL, zinc was found ensheathing the photoreceptor cell bodies, with low signal corresponding to these cell bodies themselves. In the RIS myoid zone, zinc seems to be associated with Müller cells interdigitations. Copper was identified in the choroid, RPE, RIS myoid region and ONL. Calcium was found with granular/punctate distribution in OLM, RIS myoid zone (possibly extracellularly), RPE and choroid (Fig. 4 and Supplementary material).

**Fig. 8:**
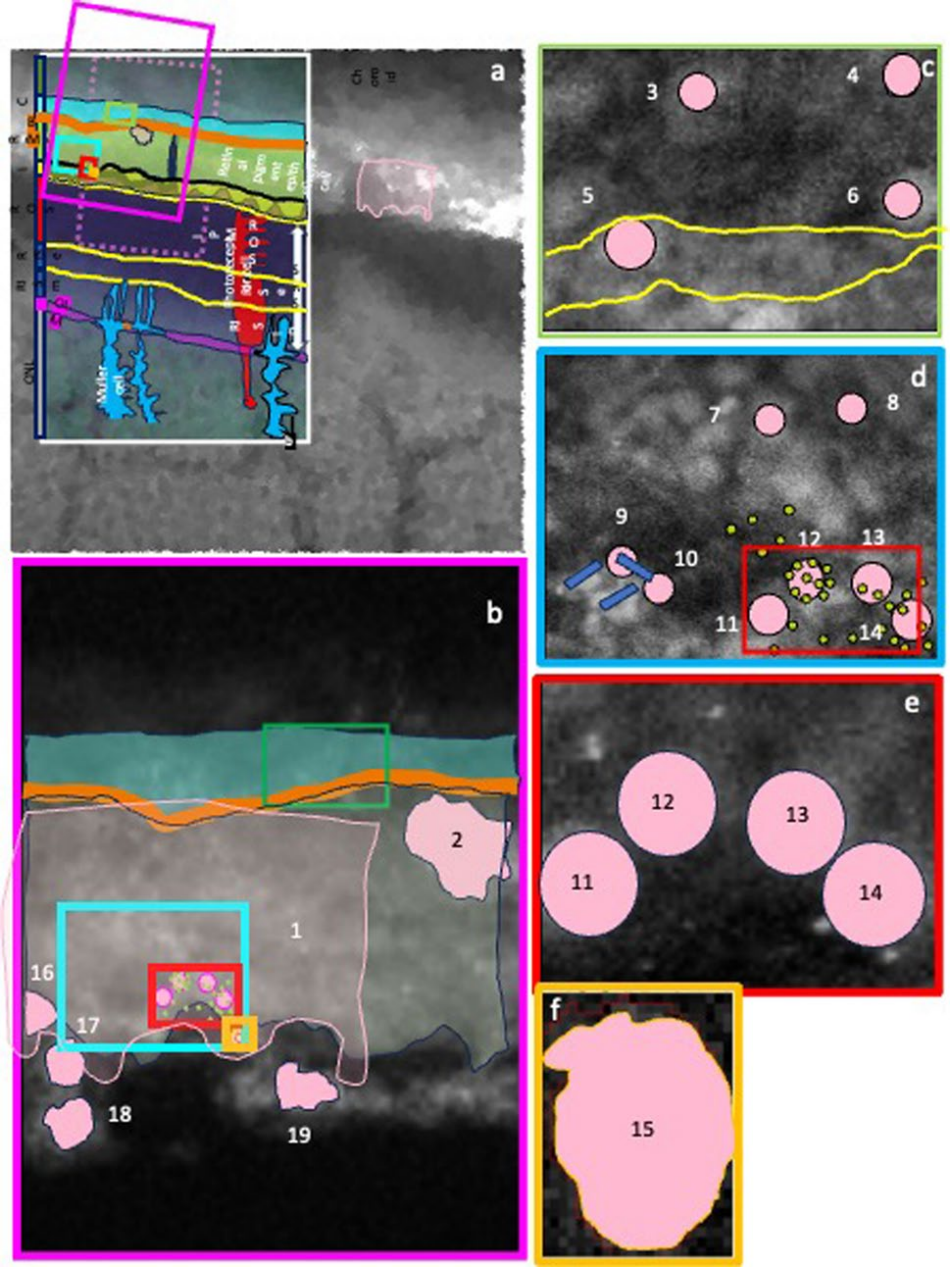
Regions of interest selected in Maps 2 (Fig, 5) **(b)**, 3 (Fig. 5) **(c)**, 4 (Fig, 5) **(d)**, 5 (Fig. 6) **(e)** and 6 (Fig. 7) **(f)** based on selenium-enriched structures (ROI 1–19) (numbers as in Fig. 9) (see Figs. 5, 6 and 7). Concentrations [mean and range (minimum - maximun) (ppm)] of 14 elements can be seen in Table 4. The element profile of the 5th (extracellular) spherical structure (ROI 15) is different to the other 4 (ROI 11–14).

The highest amounts of the metalloid selenium (Se) were found in the inner choroid, CC, BM, RPE cells (basal, central, apical), and at the interface between the RPE interdigitations and ROS tips (Figs. 4, 6 and 7). No detectable signal was found in the remaining layers. High resolution Maps 5 (Figs. 5e and k and 6) and 6 (Figs. 5f and l and 7) revealed selenium-rich spherical structures (approximately 1 μm in diameter) at the level of the RPE apical ID intracellularly (using potassium as a reference for intracellular environment) (Fig. 6) and at the interface between the interdigitations and ROP tips. “Hot spots” of zinc with concentrations as high as 298 ppm were seen surrounding intracellular selenium containing spherical structures (Figs. 6 and 8). The selenium-rich spherical structure at the interface between the RPE apical interdigitations and ROS (Fig. 7) appeared within a membrane-like zinc-containing layer, Manganese, calcium, phosphorus and chlorine were also contained within the zinc layer. The RPE cell body (Fig. 5e and j), basal side and BM/CC (Fig. d, Fig. 5i) also contained selenium-rich spherical structures, which do not seem to respect the outer BRB (basal RPE/BM complex). Some of them colocalised with calcium.

Manganese, barium, strontium and rubidium were mainly detected in the inner choroid and RPE (Fig. 4 and Supplementary material). Barium and strontium distribution resembled calcium, that of rubidium resembled potassium (Fig. 4 and Supplementary material), while bromine distribution was similar to chlorine, with highest concentrations in choroid and ONL.

ROI in Maps 2–6 (Figs. 5, 6 and 7) based on selenium-rich structures are marked in Fig. 8. The concentration of biometals, iron, zinc, copper, manganese, calcium and the metalloid selenium (in ppm) in these 19 ROIs are presented in Table 4. RPE cell layer mean concentrations (ppm) of iron 76.1, zinc 51.3, copper 5.2, calcium 1106.8 and selenium 2.5. Subcellular 1 μm long iron enriched rods with mean concentration 363.8-427.4 ppm (ranges 88.5-492.6) could be seen cladding to selenium containing structures within the RPE apical part (Supplementary material).

.

**Table 3:** Mean and range (minimum-maximum) Ppm concentrations of barium (Ba), bromine (Br), calcium (Ca), Chlorine (Cl), copper (Cu), iron (Fe), potassium (K), manganese (Mn), phosphorus (P), rubidium (Rb), sulphur (S), selenium (Se), strontium (Sr) and zinc (Zn) in different outer retinal complex layers of 3-week-old, light-adapted, male C57BL6 mouse. Layers can be seen delineated in map 1, fig. 4 and supplementary material. Detection limit. ID, interdigitations. OLM, outer limiting membrane. ONL, outer nuclear layer. RIS, photoreceptor inner segments. ROS, photoreceptor outer segments. RPE, retinal pigment epithelium.

	INNER CHOROID	CHORIO- CAPILLARIS	RPE/BM complex	RPE ID	ROS	RIS ELLIPSOID ZONE	RIS MYOID ZONE	OLM	ONL
**Ba**	26.0 **(**< DL −81.01)	35.7 (5.72–82.34)	31.5 (3.73–97.96)	23.6 (1.24–74.88)	9.0 (< DL −23.48)	8.9 (< DL −17.68)	9.1 (< DL −16.68)	9.0 (1.91–18.48)	8.9 (< DL-20.35)
**Br**	11.3 (5.77–17.44)	14.6 (9.96–20.43)	8.5 (3.28–16.12)	5.8 (3.06–9.38)	2.9 (1.44–5.36)	4.0 (1.75–5.82)	4.9 (2.65–6.51)	5.2 (2.63–6.62)	5.9 (1.39–8.61)
**Ca**	576 (21-1918)	1101 (323–2090)	1134 (257–2503)	249 (57-1338)	95 (21–633)	170 (30–810)	245 (36-1370)	159 (49–962)	69.05 (< DL −774)
**Cl**	5989 (3950–8735)	7641 (5174–10094)	4023 (2072–7145)	2335 (845–3917)	2157 (799–3835)	3175 (1454–4983)	4135 (2460–5741)	4663 (3233–6119)	5516 (661–7970)
**Cu**	2.29 (< DL −8.25)	4.3 (1.86–9.73)	4.3 (1.35–10.95)	1.94 (< DL −9.78)	1.27 (< DL −6.01)	3.31 (< DL −7.33)	4.86 (1.25–10.24)	2.73 (< DL −8.06)	1.02 (< DL −6.53)
**Fe**	31.33 (9.87-149.43)	59.5 (35.9-172.27)	52.1 (23.78–142.8)	24.3 (7.37–68.19)	11.9 (4.22–70.77)	25.2 (10.13–52.41)	37.5 (13.95–68.34)	31.1 (10.37–109)	11.15 (2.28-168.35)
**K**	4748 (2532–9070)	6994 (3944–10575)	6169 (2534–10740)	2653 (771–7598)	1912 (780–3310)	2670 (1439–3929)	3544 (2351–4609)	4045 (2725–5182)	4874 (521–6775)
**Mn**	0.8 (< DL −4.3)	1.1 (< DL −3.85)	1.4 (< DL −4.65)	0.5 (< DL −3)	0.4 (< DL −2.34)	0.4 (< DL −2-32)	0.5 (< DL −2.7)	0.6 (< DL −2.6)	0.5 (< DL −2.63)
**P**	2010 (< DL −9116)	3431 (1.17–10514)	7946 (415-17095)	8020 (1865–15600)	8541 (1953–16270)	9475 (2600–16354)	9503 (2983–18338)	10,878 (3932–22398)	21,074 (1160–41903)
**Rb**	4.5 (1.03–10.39)	6.4 (2.73–11.16	4.8 (1.04–10.13)	3.4 (< DL −6.76)	1.4 (< DL −3.34)	1.8 (< DL −3.04)	2.3 (1.14–3.61)	2.5 (1.34–3.58)	3.4 (< DL −5.53)
**S**	4114 (1033–8631)	7177 (3359–10450)	7601 (4745–11067)	5829 (2177–10375)	4777 (1621–8203)	5559 (2404–8137)	5941 (3425–9037)	5501 (3401–8248)	3698 (203–7372)
**Se**	0.8 (< DL −3.9)	1.8 (< DL −4.7)	2.4 (< DL-8.2)	1.3 (< DL −6.6)	0.5 (< DL −1.2)	0.5 (< DL −1.0)	0.6 (< DL −1.2)	0.7 (< DL −1.23)	0.5 (< DL −2.2)
**Sr**	3.137 (< DL −8.93)	4.492 (1.5–9.05)	3.583 (< DL −8.56)	2.498 (< DL −5.93)	1.067 (< DL −2.06)	1.067 (< DL −2.01)	1.087 (< DL −2.12)	1.039 (< DL −1.72)	0.915 (< DL −1.85)
**Zn**	21.4 (4–59)	45 (27.8–77)	42 (17–73)	18.6 (3.6–54.1)	9.1 (2.6–22.2)	16 (7.4–29)	24.8 (< DL −36)	31.8 (< DL −44.1)	26.2 (3.2-51-8)

**Table 4:** Mean (range) ppm concentrations of iron, zinc, copper, calcium, manganese and selenium in manually selected 19 ROIs within the RPE, at the RPE cell basal part, bruch´s membrane (BM) and choriocapillaris (CC) complex, RPE interdigitations and apical interface with ROS tips, as delineated in fig. 8. The rois were selected in map 2 (Fig, 5), map 3 (Fig. 5), map 4 (Fig. 5), map 5 (Fig. 6) and map 6 (Fig. 7) based on high selenium content.

	Fe	Zn	Cu	Ca	Se
**RPE cell** **(ROI 1)**	76.1 (29.1-318.8)	51.3 (28.2–76.8)	5.2 (2.2–10.1)	1106.8 (263.0-2068.0)	2.5 (0.8–7.6)
**Subcellular structure within the RPE** **(ROI 2)**	85.0 (54.5-123.6)	54.3 (38.5–70.9)	5.9 (3.1–9.2)	1049.5 (655.0-1478.9)	3.7 (1.5–5.9)
**CC/BM complex** **(ROI 3)**	19.1 (14.1–27.7)	31.4 (27.3–35.3)	3.7 (2.4–4.8)	695.1 (500.2–1089.0)	3.6 (2.6–4.5)
**CC/BM complex** **(ROI 4)**	20.1 (12.7–45.4)	32.8 (29.9–36.3)	4.1 (2.6–5.9)	681.3 (500.0-1090.0)	3.0 (2.3-4.0)
**RPE basal side/BM complex** **(ROI 5)**	26.9 (18.0-47.8)	29.4 (26.5–31.9)	4.6 (3.5–5.6)	532.3 (500.5-588.5)	2.8 (2.1–3.2)
**RPE basal side/BM complex** **(ROI 6)**	19.5 (15.2–25.6)	40.3 (37.4–42.6)	4.2 (3.3–5.2)	547.4 (501.0-631.2)	3.5 (2.7–4.2)
**Within the RPE** **(ROI 7)**	85.0 (54.5-123.6)	54.3 (38.5–70.9)	5.9 (3.1–9.2)	1049.5 (655.0-1478.9)	3.7 (1.5–5.9)
**Within RPE** **(ROI 8)**	77.4 (44.9-297.7)	59.9 (47.7-69.34)	5.1 (3.4-7-7)	1352.5 (925.2–1834.0)	2.1 (1.1–3.8)
**Within RPE** **(ROI 9)**	81.3 (68.2-100.8)	51.8 47.2–57.8)	12.3 (8.0-15.9)	1285.4 (1096.7-1480.6)	2.8 (1.8-4.0)
**Within RPE** **(ROI 10)**	83.5 (69.8-147.3)	86.9 (75.4–93.7)	9.2 (6.0-12.2)	1668.5 (1322.5-2033.9)	5.5 (3.6–7.2)
**Se sphere 1** **(ROI 11)**	23.7 (16.7–31.4)	41.3 (35.1–65.2)	6.4 (3.6–8.6)	1056.9 (618.6-1754.7)	5.6 (2.2–8.1)
**Se sphere 2** **(ROI 12)**	30.4 (19.0–89.0)	52.7 (34.9–64.2)	7.0 (4.7–9.7)	1236.8 (778.6-1786.5)	4.7 (2.3–6.8)
**Se sphere 3** **(ROI 13)**	31.0 (24.3–61.1)	51.2 (33.7-295.2)	7.1 (4.2–10.1)	1309.3 (743.4-1776.7)	4.2 (2.2-6.0)
**Se sphere 4** **(ROI 14)**	26.7 (21.0-37.6)	42.6 (32.2-104.6)	5.8 (3.5–8.2)	1161.1 (595.5-1941-3)	4.6 (2.0-6.6)
**Se sphere 5** **(ROI 15)**	20.4 (14.9–26.5)	43.5 (26.8-70-7)	4.7 (2.8-7.0)	7449.4 (1517.5-18092.9)	3.4 (1.5–5.7)
**RPE-ROS interface** **(ROS 16)**	78.0 (54.4-100.2)	63.3 (48.4–73.8)	5.7 (4.6–7.5)	1051.7 (803.09-1213.6)	5.7 (3.6–7.5)
**RPE-ROS interface** **(ROI 17)**	64.5 (48.6–86.2)	46.6 (33.1–51.7)	5.1 (2.8–7.3)	530.3 (287.0-1102.5)	4.5 (2.6–5.6)
**RPE-ROS interface** **(ROI 18)**	40.2 (21.5–55.1)	33.9 (17.7–47.4)	3.1 (1.4–5.4)	290.4 (137.2-435.8)	3.3 (1.2–5.5)
**RPE-ROS interface** **(ROI 19)**	49.5 (31.7–61.6)	44.3 (28.2–56.7)	4.7 (2.6–6.6)	405.2 (264.0-524.7)	5.6 (2.3–8.5)

## Discussion

The light-adapted outer retinal complex 2D maps and 3D surface plots, of the 14 elements, showed consistent and significant heterogeneity in the elemental composition of specific layers and subcellular structures. This is presumed to be related to their various constituents, compartments (i.e. intra and extracellular spaces, cell membranes) and functions (Table 5).

Chlorine, the constituent of chloride (Cl^−^), principal extracellular anion involved in fluid, electrolyte, acid-base balance and osmotic pressure^47,48^, was found in high amounts in the choroid. There was a sharp drop at the basal RPE/BM interface, which is known to have active transport mechanisms for chloride ions^49^. Potassium, the main intracellular cation involved in acid-base balance, intracellular osmotic pressure, membrane conduction and function, protein synthesis, glycogenesis and energy metabolism^50^, was found in high amounts within the RPE and ONL cell bodies of photoreceptors (Fig. 4 and Supplementary material). Phosphorus, a constituent of phospholipids (e.g. phosphatidylserine), phosphoproteins, nucleic acids, phosphorylated metabolic intermediates, and the high energy compound, ATP^51^, was found in high amounts in ONL photoreceptor cell bodies, RIS and ROS (particularly their tips). Sulphur, the element found in the aminoacids cystine, cysteine and methionine^52^, whose primary function is the dynamic regulation of proteins structure and function^53^, was found at the highest concentration in layers corresponding to CC, RPE and ROS tips (Fig. 4 and Supplementary material).

The distribution of zinc, iron and copper in mouse, is similar to other mammals (i.e. rat and monkey)^1–3^. Iron contained in heme of red blood cells and within macrophages may have contributed to the signal in the form of “hotspots” in the choroid. In the RIS layer, iron may be within mitochondria, involved in transfer of electrons and in respiration^54^. A cluster of 1 μm long rod-like structures appeared intracellularly in the apical part of a RPE cell “cladding” a selenium-containing structure. (Fig. 8 and Supplementary material).

Zinc was predominantly localised to the choroid, RPE cell body and interdigitations, RIS myoid zone, OLM and ONL. Zinc distribution resembled the immunolabelling of the membrane zinc metalloprotein, carbonic anhydrase XIV in Mūller cells, and RPE apical and basolateral membranes^55^. Functions of zinc in these locations might be (Table 5): (a) to alter the behaviour of membrane constituents (e.g. ion channels, ion transporters, receptors) and/or (b) to combat lipid peroxidation and/or antioxidant mechanisms. Putative mouse retinal zinc-binding proteins are involved in the following biological processes: (1) localization to the cell, (2) protein-containing complex subunit organisation and assembly, (3) RNA processing, (4) protein translation, (5) aminoacid activation and (6) cellular component biogenesis^56^.

Copper was identified in the choroid, RPE, RIS myoid region and ONL. Copper is important for all oxygen-requiring processes. It is involved in energy and iron metabolism. It is an essential cofactor and binds to enzymes (e.g. superoxide dismutase, tyrosine hydroxylase, cytochrome c oxidase, a very important mitochondrial energy production, electron transport and proteins)^57^.

Calcium was found, with granular punctate distribution, in elevated amounts at the level of the choroid, RPE, and RIS myoid zone (possibly extracellularly) and OLM. Calcium plays a crucial role in numerous retinal physiological processes. It is related to membrane permeability, enzyme activity, intercellular communication, phototransduction, integrin function, RPE microvilli function, ionic function and physiochemical properties (i.e. structural integrity and viscosity) of the IPM)^58^.

Bromine distribution was similar to chlorine (highest concentrations in choroid and ONL). Manganese, strontium and rubidium were detected in the choroid and RPE. Strontium and barium distribution resembled calcium, while that of rubidium resembled potassium. This is consistent with previous studies showing high metabolic interchangeability between rubidium and potassium^59^. strontium and calcium^60^; and barium and calcium^61^. The function of Barium in the outer retinal complex is not fully understood^62–69^. It seems to be involved in membrane channels function.

The highest amounts of the metalloid selenium were found in the inner choroid, CC, BM, RPE cells (basal, central, apical), and at the interface between the RPE interdigitations and ROS tips (Figs. 4, 6 and 7). Quantification within the RPE up to 8.5 ppm, revealed the ability of these cells to handle high amounts (potentially toxic). For comparison, studies in non-ocular tissues have reported much lower values for selenium (in ppm wet weight) cardiac muscle, 0.15 to 0.20; pancreas, 0.34 to 0.44; ovary, 0.19 to 0.25; cerebrum, 0.07 to 0.09^70^. Selenium has unique optical and physicochemical properties^71–82^ of nucleophilicity, ionization and redox potential. It behaves differently in light and dark. It combines easily with other elements to form selenides (e.g. manganese selenide) and selenium-calcium phosphate biomineral. All these properties are likely to be beneficial for retinal functions.

We identified for the first time, selenium-rich spherical structures (approximately 1 μm in diameter) in the CC, RPE cell body, RPE apical interdigitations and RPE/ROS interface. In the latter, within a membrane-like zinc-containing layer, also enclosing manganese, calcium, phosphorus and chlorine.

Based on our findings, it is impossible to establish the element-containing compounds and/or to assign any precise physiological significance to the elements themselves. We can only speculate in the context of existing evidence in the literature (Table 5). Some of the selenium-containing structures identified in the centre of the RPE and choroid could potentially be melanosomes^83,84^. However, we consider the 1 μm sphere-shaped structures are unlikely to represent melanosomes or a selenium repository. They resemble selenium particles observed in bacteria^85–87^, thought to be composed of interconnected nets of elemental selenium and/or selenium containing protein(s), whose biological processes include: (1) cellular redox homeostasis and (2) hydrogen peroxide metabolic process. Various oxidoreductases and antioxidant selenoproteins have been identified in the retina (i.e. thioredoxin reductase, glutathione peroxidase and methionine sulfoxide reductase)^88–90^. Selenoproteins contain the genetically encoded residue, selenocysteine with a selenium atom, in place of sulphur in cysteine. It is structurally similar to cysteine but has lower redox potential and higher catalytic activity^91^.

**Fig. 9:**
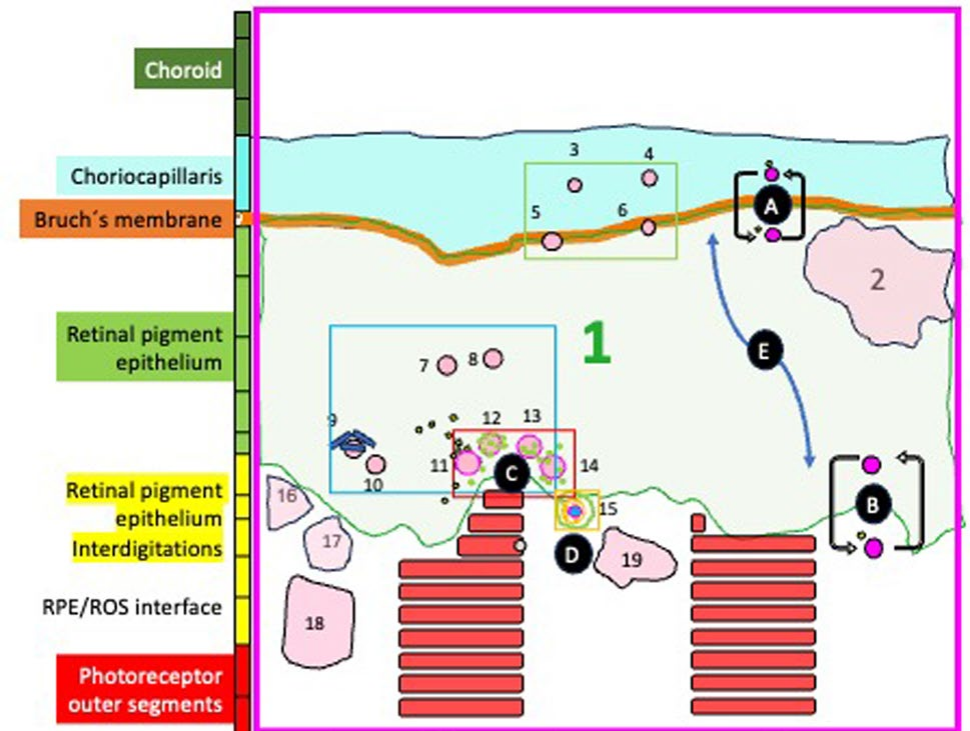
Schematic representation showing hypothesized components of selenium biology in the RPE basal, central and apical regions, based on our findings in this study and evidence in the literature. We propose there is a dedicated selenium-containing structure with the ability to cross RPE cell membranes (i.e. the outer BRB) at the basal **(A)** and apical side **(B)**, with potential biological function(s). Selenium rich spherical structure at the RPE/photoreceptor interface and RPE apical interdigitations might be involved in: (a) photoreceptor-OS phagocytosis **(C)**, (b) RPE microvilli membrane vesiculation **(D)** and/or (c) trans-RPE transport **(E)**. Selenium-rich spherical structures at the RPE apical side could be a regulatory factor or vehicle to shift substrates between the RPE and photoreceptors **(C**,** D)**, while allowing chemical and physical reactions exchanging electrons and offering antioxidant protection. “Hot spots” of zinc with concentrations as high as 298 ppm were seen surrounding intracellular selenium containing spherical structures (yellow dots). The selenium-rich spherical structure at the interface between the RPE apical interdigitations and ROS (Fig. 7) appeared within a membrane-like zinc-containing layer (ROI 15), Manganese, calcium, phosphorus and chlorine were also contained within the zinc layer. We speculate a zinc-layered vesicle containing selenium-rich spherical structures, calcium, phosphorus, manganese and chlorine is either emanating from the apical part of the RPE or being ingested. Zipping seems to be carried out by a zinc-rich structure (possibly a zinc-binding protein) (green dots). Subcellular 1 μm long iron enriched rods (blue rods) with mean concentration 363.8-427.4 ppm (ranges 88.5-492.6) could be seen cladding to selenium containing structures within the RPE apical part The RPE cell body (Fig. 5e and j), basal side and BM/CC (Fig. d, Fig. 5i) also contained selenium-rich spherical structures, which do not seem to respect the outer BRB (basal RPE/BM complex). Some colocalised with calcium (ROI 3 and ROI 4) The role, mechanism, and metabolism of selenium in the outer retinal complex of C57BL6 mice remain to be fully identified.

We hypothesise there is a dedicated selenium-containing structure in the outer retinal complex with the ability to cross RPE cell membranes (i.e. the outer BRB) and with potential biological function(s) (Fig. 9). The various arrangements of these well-structured selenium-rich spherical structures could represent coordinated physicochemical changes with trace element exchanges during highly regulated biological process(es). Selenium-containing spherical structures in the RPE apical interdigitations and RPE/ROS interface might be involved in photoreceptor-ROS phagocytosis, RPE microvilli membrane vesiculation and/or trans-RPE transport. They could be a regulatory factor or vehicle to shift substrates between the RPE and photoreceptors. while allowing chemical and physical reactions (e.g. exchanging electrons and offering antioxidant protection). We speculate a zinc-layered vesicle containing selenium-rich spherical structures, calcium, phosphorus, manganese and chlorine is either emanating from the apical part of the RPE or being ingested. Zipping seems to be carried out by a zinc-rich structure (possibly a zinc-binding protein).

In this study, we demonstrate the capability and utility of nanobeam synchrotron X-ray fluorescence microscopy in investigating the elemental composition of the outer retinal complex at the tissue, cellular, and subcellular levels, using fresh snap-frozen and air-dried thin cryosections. These findings set the baseline for further investigations of metal(loid) biology research in the outer retinal complex, in physiological and pathological conditions.

### Limitations of the study

Due to the limited beam time available, the scanning step size was adjusted to strike a balance between the target resolution at best matching the focal spot size. Smaller steps would have entailed longer measuring times. Another limitation of this study is that it is based on elements distributions and correlations. We do not imply any element-related retinal functions.

### Further studies

Since the first publication of retina synchrotron XRF microscopy nearly 40 years ago^92^, synchrotron X-ray fluorescence microscopy is developing very fast, expanding its capabilities. Future studies looking at interlinked applications of X-ray fluorescence microscopy with genetics and expression of proteins will help us expand our knowledge of molecular and cellular processes controlling metal(loid) homeostasis (i.e. transporters, routes of entry and movement across compartments in the outer retina) and their precise role in retina physiology and disease.

The therapeutic use of selenium nanoparticles (e.g. selenium-calcium phosphate biomaterials) is currently being investigated in Alzheimer disease, diabetes, rheumatoid arthritis, and cancer^93–96^. Selenium nanoparticles have small size, low toxicity, strong biocompatibility, antioxidant and anti-inflammatory properties. They could potential be used using the endogenous selenium homeostatic pathways in the outer retina complex. Future research in this field might open numerous opportunities in choroidal and retinal practical therapeutic implications.

**Table 5:** Summary of nanobeam synchrotron XRF microscopy distribution of iron, zinc, copper, calcium, manganese and selenium in adult mouse light adapted outer retinal complex (from this experiment), in the context of the literature evidence on putative element-containing structures, retinal metal binding proteins, their molecular functions and biological processes^56^.

	ZINC	COPPER	CALCIUM	IRON	SELENIUM	MANGANESE
**OUTER RETINAL LAYER LOCALISATION**	Choroid RPE RIS OLM ONL	Choroid RPE b RIS ONL	Choroid RPE RIS	Choroid RPE RIS OLM ONL	Choroid RPE RPE/photoreceptor interface	Choroid RPE RIS ONL
**METAL CONTAINING STRUCTURE**	Membrane proteins	Mitochondrial respiratory proteins	Second messenger	Iron rods Fe-S clusters	Selenium microspheres	
**PUTATIVE** **METAL BINDING PROTEINS** ^56^	Rhodopsin Carbonic anhydrase XIV Retinol dehydrogenase Rod GMP phosphodiesterase beta Calbindin D28	Cytochrome oxidase Superoxide dismutase	Recoverin GCAP1 Calmodulin Phosphodiesterase 1B Calcium-binding protein 4 Calcium-binding mitochondrial carrier protein SMaMC-2 Rotamase Reticulocalbin 1 Lysophosphatidylcholine acyltransferase 1, Mitochondrial glycerol 3-phosphate dehydrogenase Fibronectin receptor subunit beta (integrin beta 1) Calbindin D28 Integrin alpha V	RPE65 Haemoglobin Ferritin Hephaestin	Thioredoxin reductase, Glutathione peroxidase Methionine sulfoxide reductase	Serine/threonine protein phosphatase PP1 alpha catalytic subunit Serine/threonine protein phosphatase PP1 beta catalytic subunit Rod GMP phosphodiesterase beta
**PUTATIVE** **METAL BINDING PROTEINS** **MOLECULAR FUNCTION** ^56^	Acid base regulation pH buffering Fluid balance Antioxidant Visual cycle	Respiration Energy metabolism	Membrane permeability Enzyme activity, Intercellular communication, Phototransduction, Integrin function RPE microvilli function Ionic function Physiochemical properties of the interphotoreceptor matrix (e.g. structural integrity, viscosity)	Transfer of electrons in mitochondria Respiration Oxygen transport	Redox biology Antioxidant	Regulation of the intracellular concentration of cAMP and cGMP
**PUTATIVE** **METAL BINDING COMPOUNDS AND PROTEINS POTENTIAL** **SPECIFIC RETINA FUNCTIONS** ^56^	Dark adaptation Regeneration of rhodopsin in RPE Light reactions in photoreceptors and Muller cells		Phototransduction	Visual cycle	Trans-RPE transport ROS phagocytosis Antioxidation Binding role Cytoskeleton function	Phototransduction

## Electronic supplementary material

Below is the link to the electronic supplementary material.

Supplementary Material 1

## Data Availability

https://zenodo.org/uploads/15705573?token=eyJhbGciOiJIUzUxMiJ9.eyJpZCI6IjJlNDVlZWQxLTJjYzItNDM2OS1iN2IyLTQ4YjAxMDgxOGIyYyIsImRhdGEiOnt9LCJyYW5kb20iOiI3YzBiZTcxN2IzYjMzMDhlMGRmNTQzYTVkMDM2MTlhNyJ9.JqNc3NpapI9wVQJiyAe4iVtqTwN9UoywOL3GdJgzi0PMf6aSD1g1EtWe9ZW-O0WNy1byLI87FMGvOdkOUFMpIAESRF Data Policy 2024 can be foundhttps://www.esrf.fr/datapolicy[2]Favre-Nicolin, V., Götz, A., Krisch, M., & Martinez-Criado, G. (2024). ESRF Data Policy 2024 (Version 1) [dataset]. European Synchrotron Radiation Facility. doi.org/10.15151/ESRF-DC-1534175008.
