# Supplementary material for: Nanoprobe synchrotron X-ray fluorescence microscopy reveals selenium-rich spherical structure in mouse retinal pigment epithelium
Source: Sci Rep. 2025 Aug 1;15:28070. doi: 10.1038/s41598-025-11678-4 (PMC12317131; doi:10.1038/s41598-025-11678-4)

MAP 1

2D Colour elemental maps
3D elemental surface plots

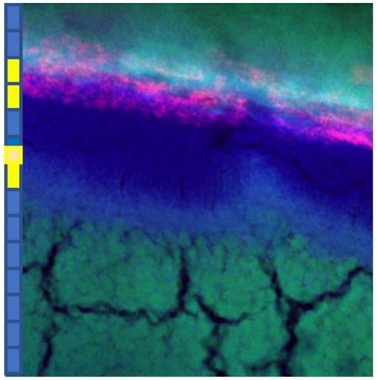

Map 1

Selenium Chloride Sulphur
(SeClS)

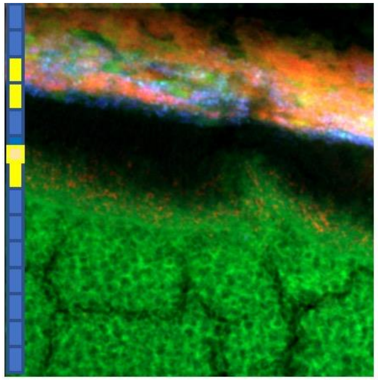

Map 1

Calcium Zinc Selenium
(CaZnSe)

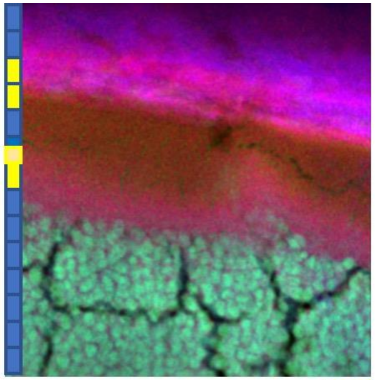

Map 1

Sulphur Phosphorus Potassium
(SPK)

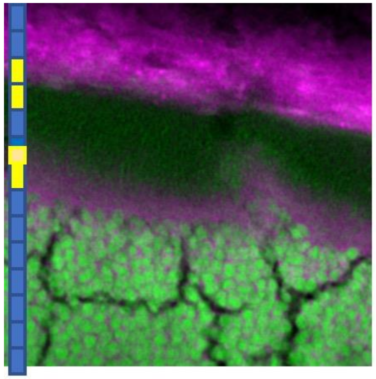

Map 1

Zinc Phosphorus
(ZnP)

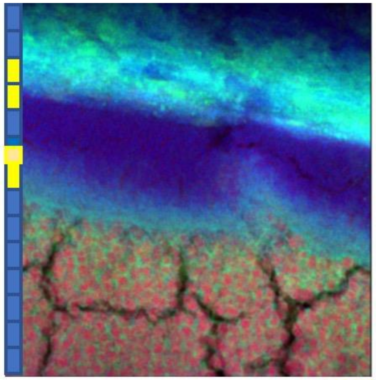

Map 1

Phosphorus Zinc Sulphur
(PZnS)

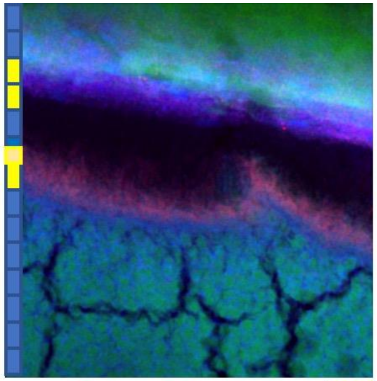

Map 1

Copper Chloride Zinc
(CuClZn)

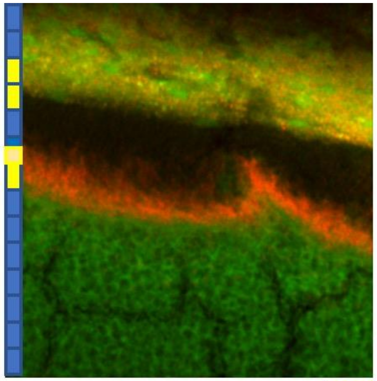

Map 1

Copper Zinc
(CuZn)

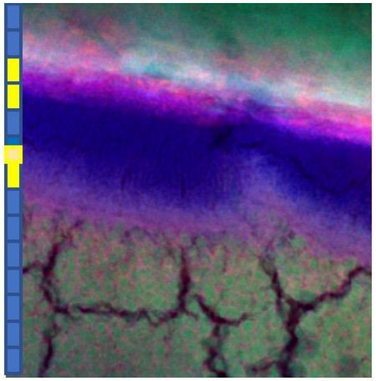

Map 1

Zinc Chloride Sulphur
(ZnClS)

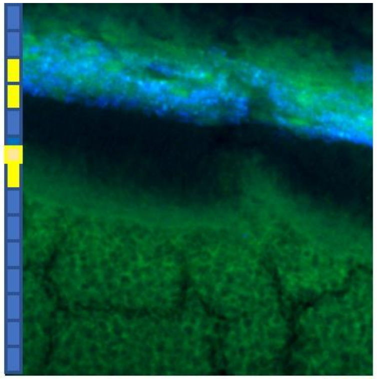

Map 1

Zinc Selenium
(ZnSe)

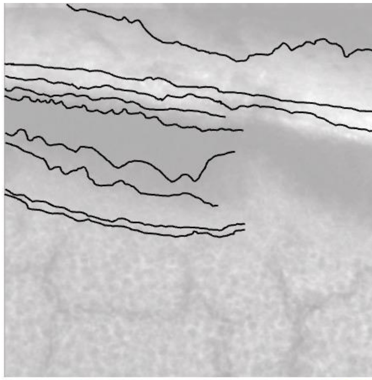

Map 1

Calcium Zinc Selenium
(CaZnSe)

MAP 1

2D Colour elemental maps

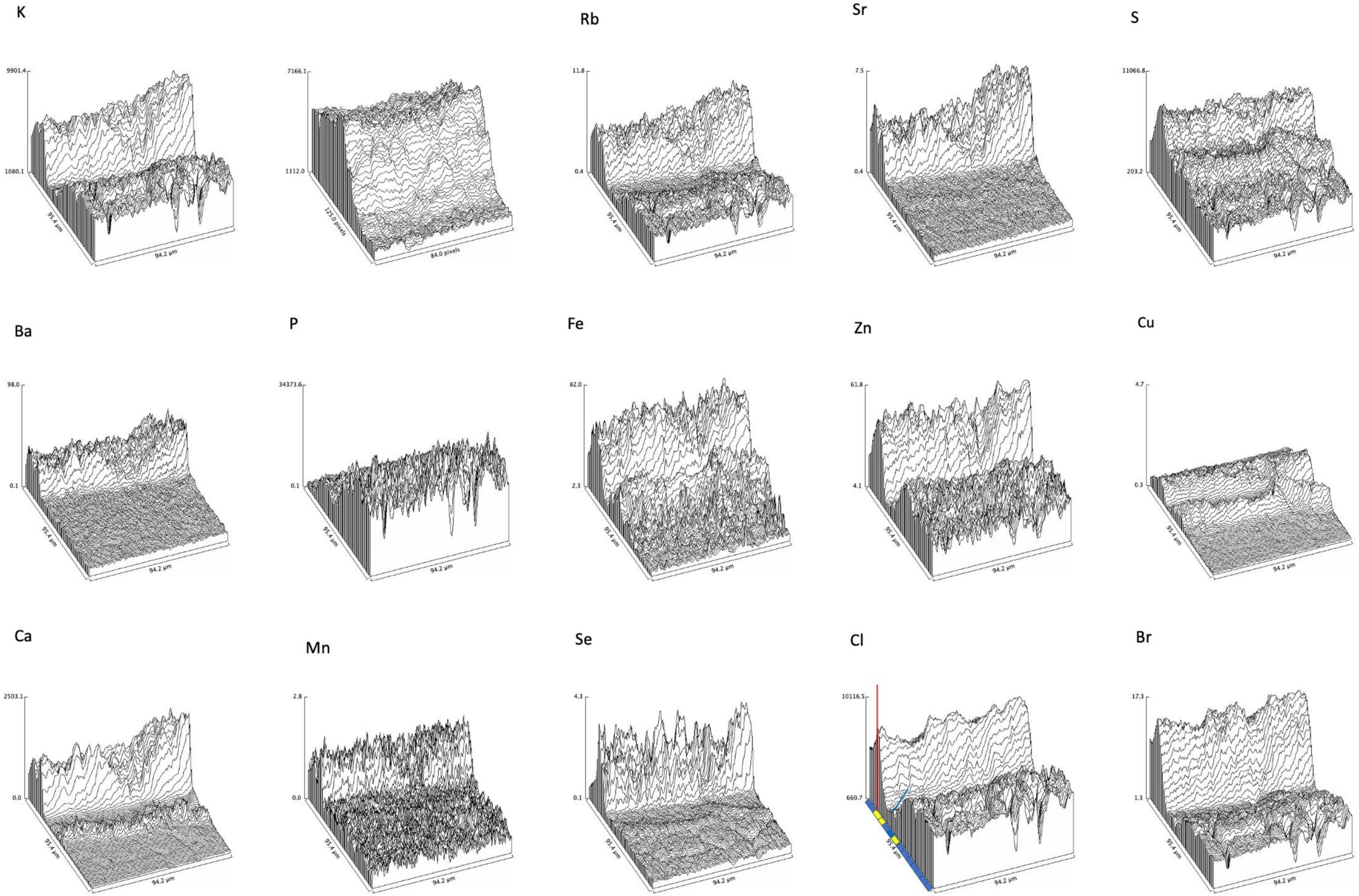

MAP 2

2D Colour elemental maps

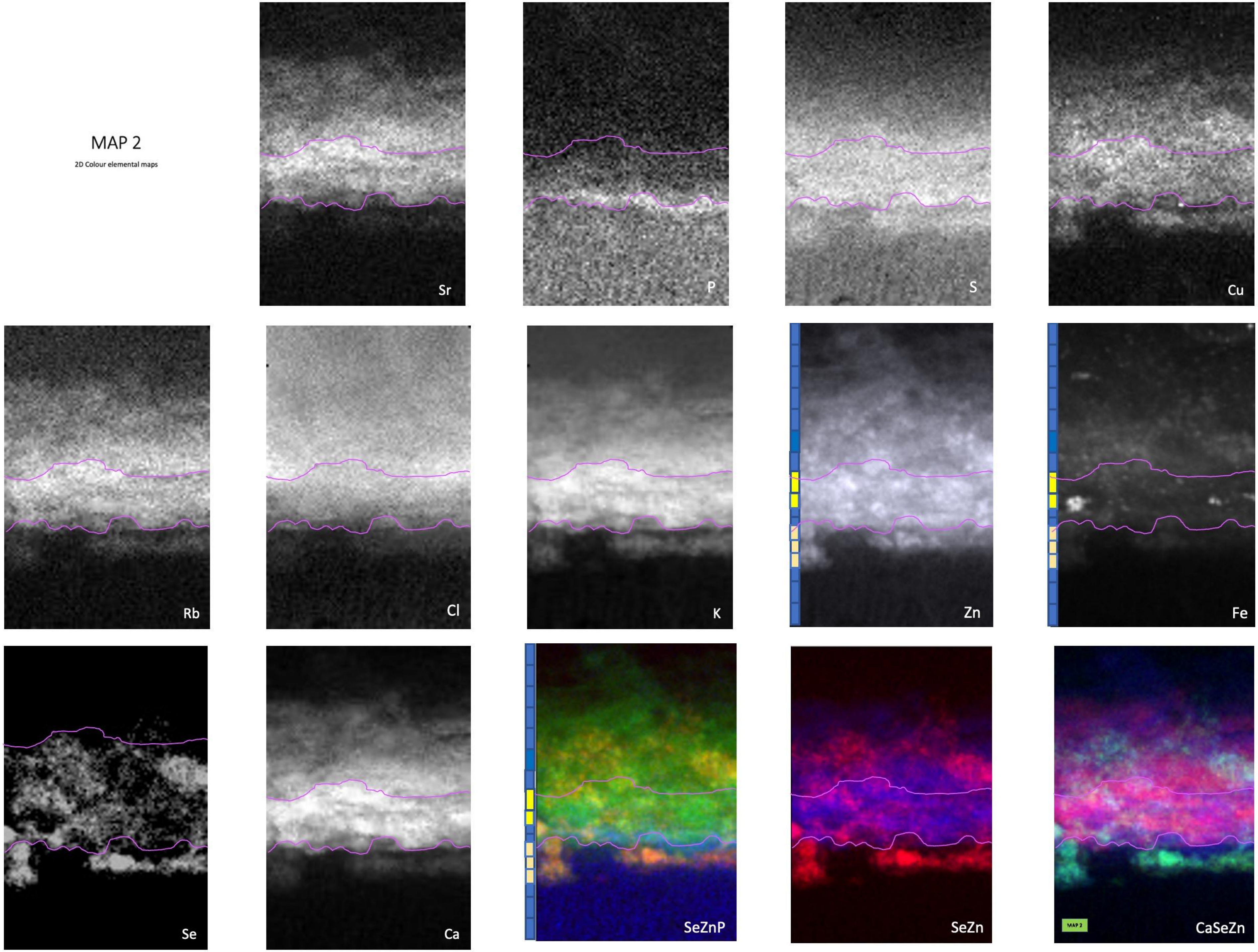

MAP 2

2D Colour elemental maps

K

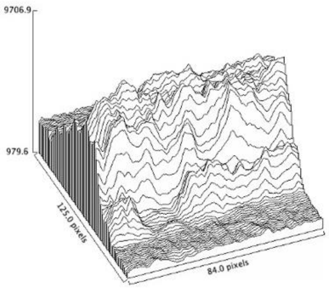

S

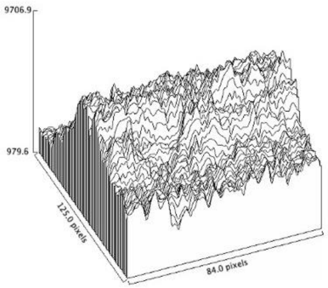

P

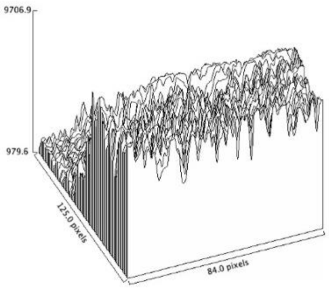

Ca

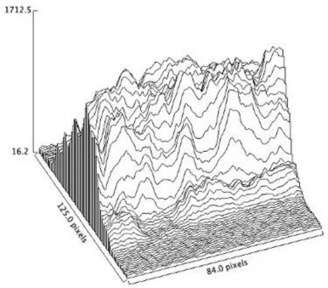

Cu

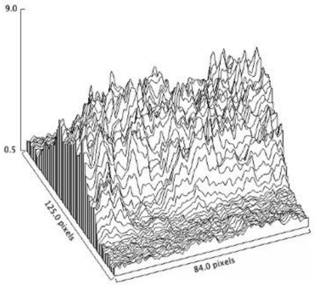

Se

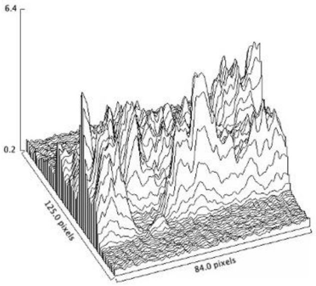

Cl

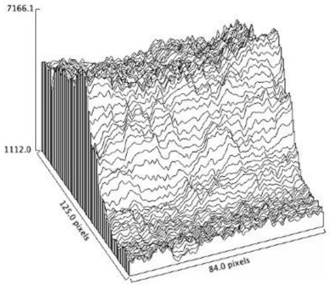

Fe

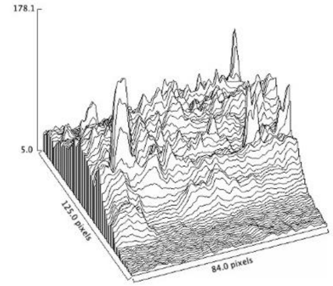

Zn

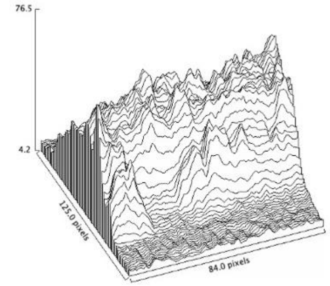

MAP 3

2D Colour elemental maps
3D elemental surface plots

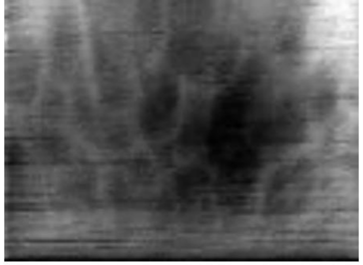

Cl

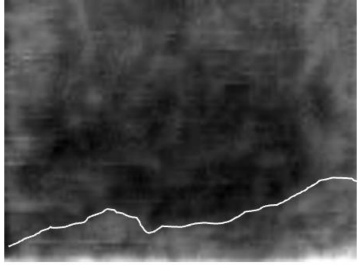

K

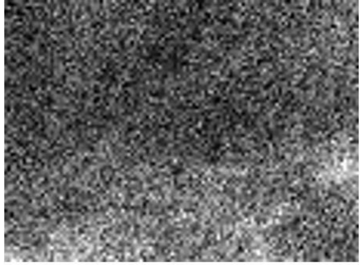

P

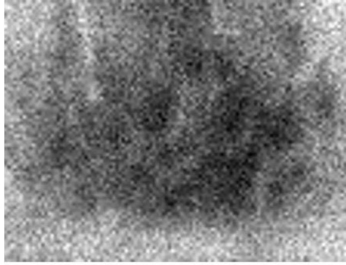

S

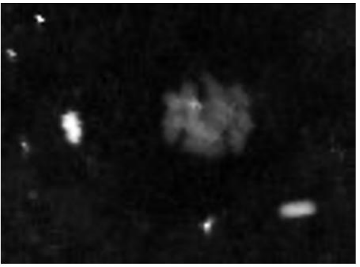

Fe

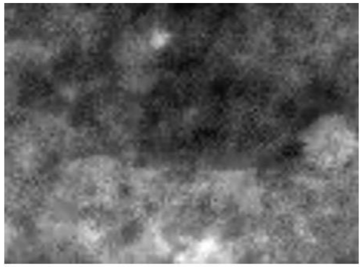

Zn

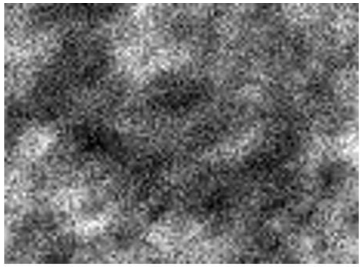

Cu

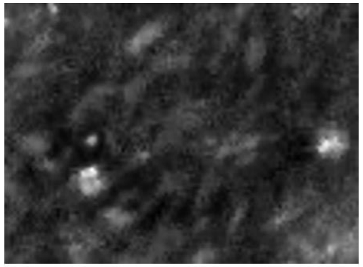

Ca

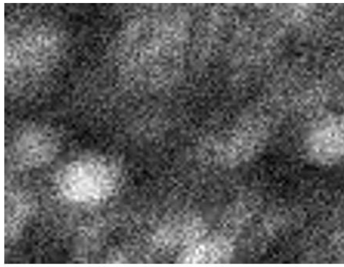

Se

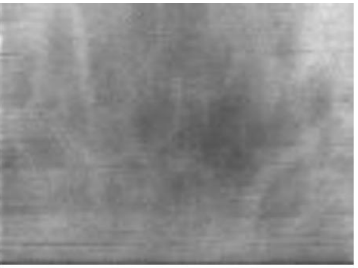

Br

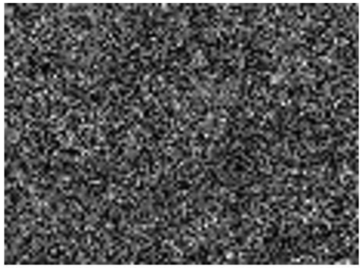

Mn

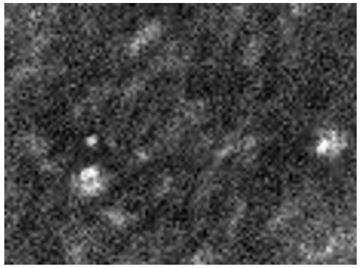

Sr

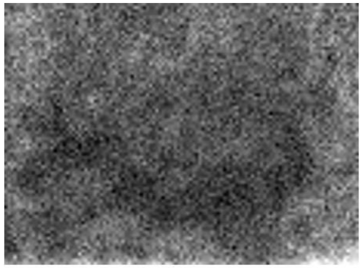

Rb

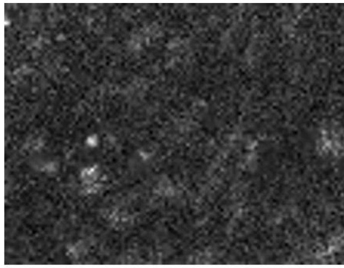

Ba

MAP 3

2D Colour elemental maps
3D elemental surface plots

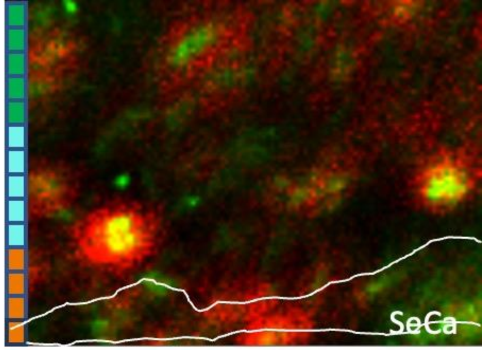

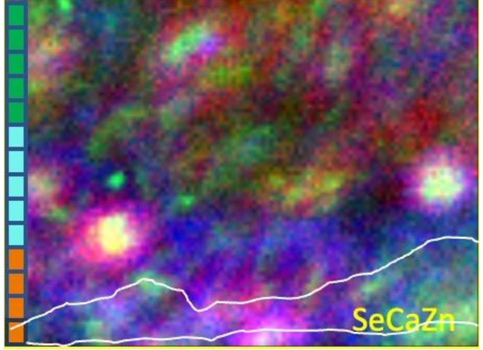

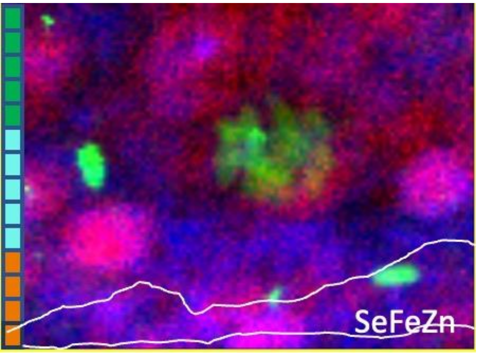

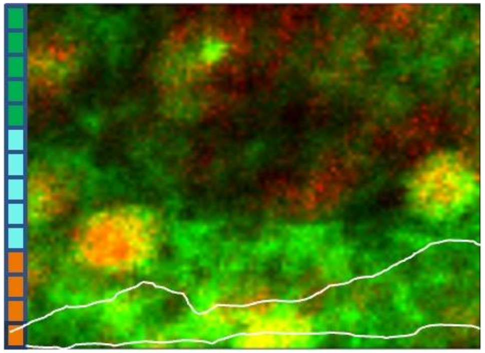

SeZn

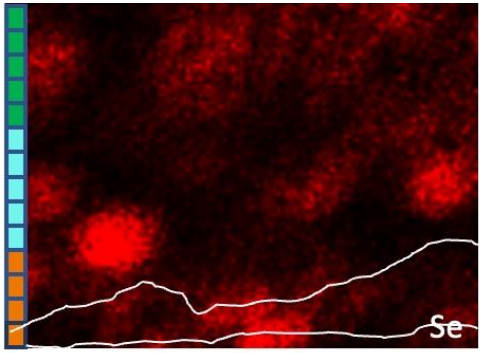

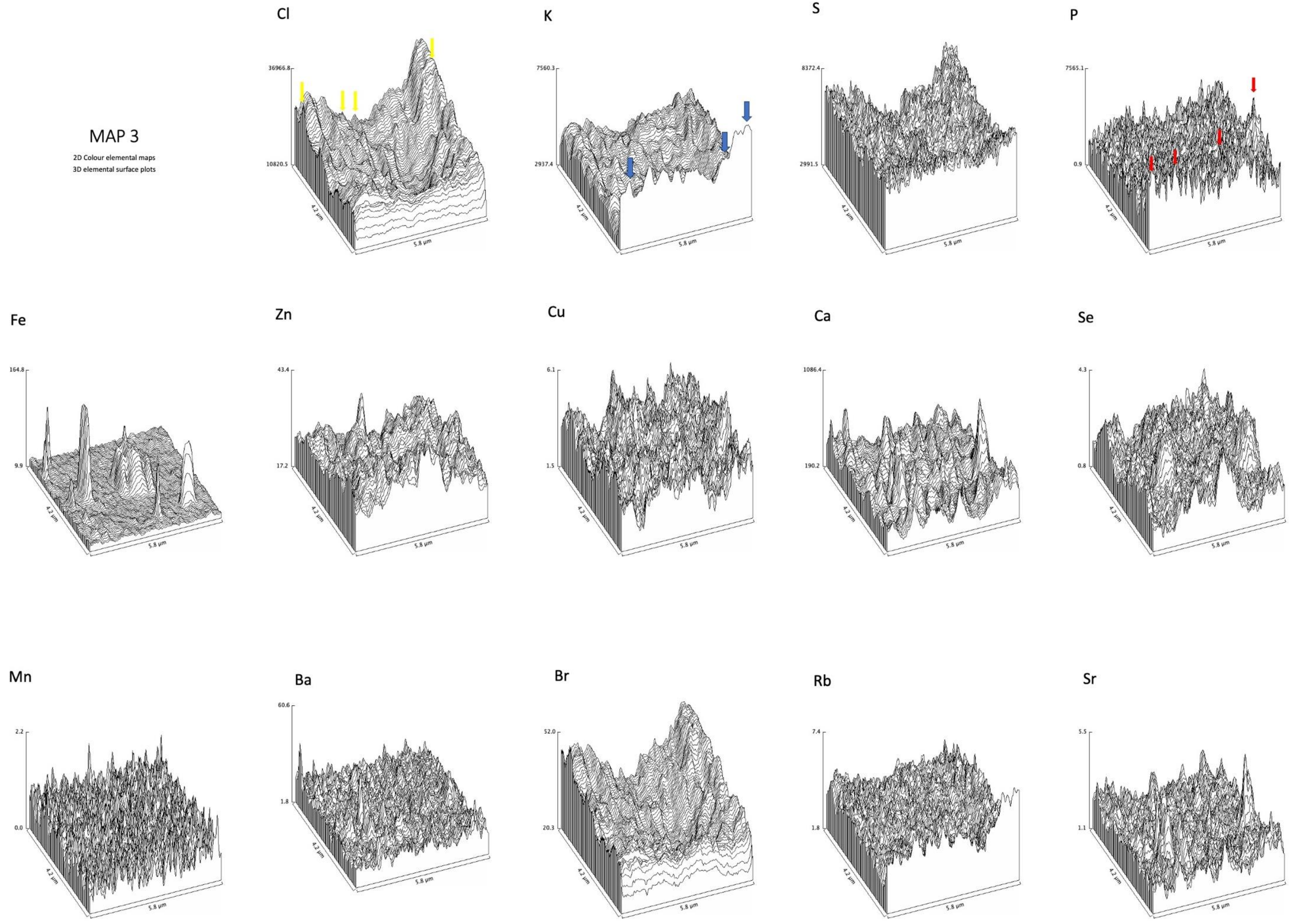
MAP 3
2D Colour elemental maps
3D elemental surface plots
Cl
36966.8
10820.5
K
7560.3
2937.4
S
8372.4
2991.5
P
7565.1
0.9
Fe
164.8
9.9
Zn
43.4
17.2
Cu
6.1
1.5
Ca
1086.4
190.2
Se
4.3
0.8
Mn
2.2
0.0
Ba
60.6
1.8
Br
52.0
20.3
Rb
7.4
1.8
Sr
5.5
1.1
4.2 µm
5.8 µm

MAP 4

2D Colour elemental maps

3D elemental surface plots

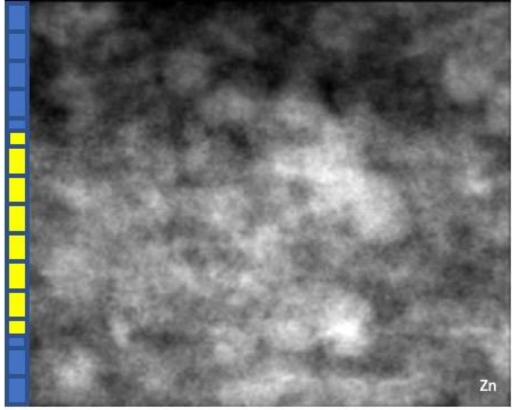

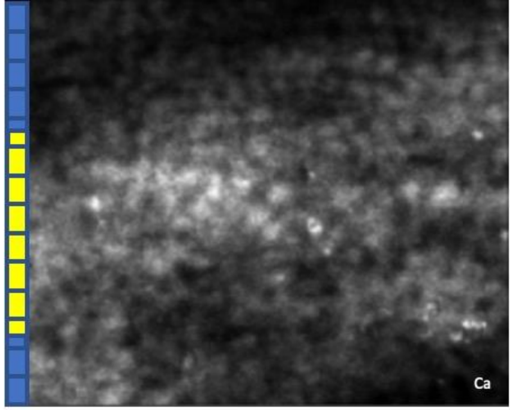

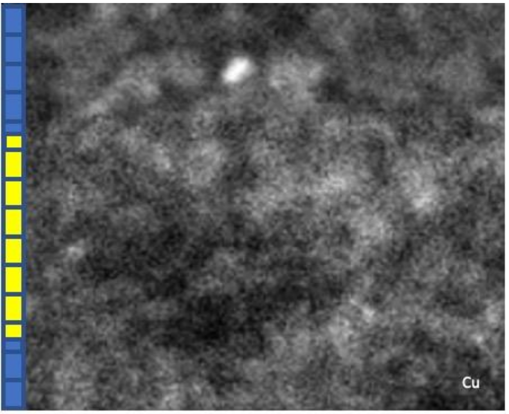

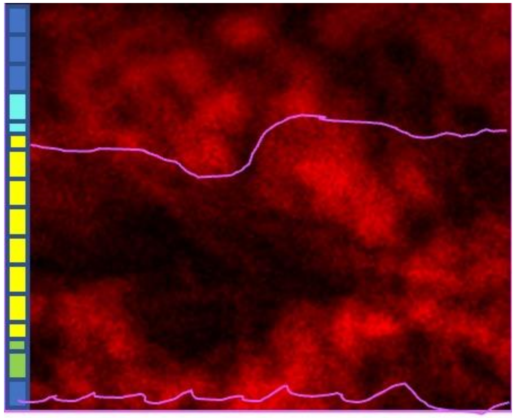

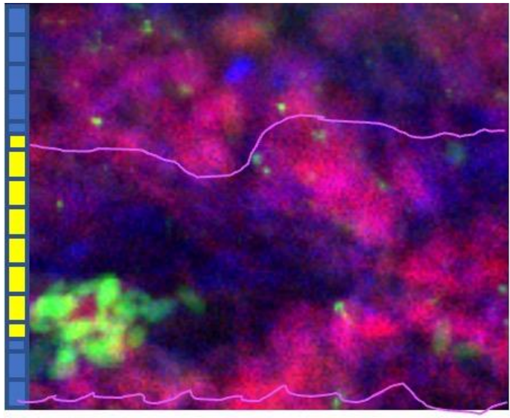

Map 4

SeFeZn

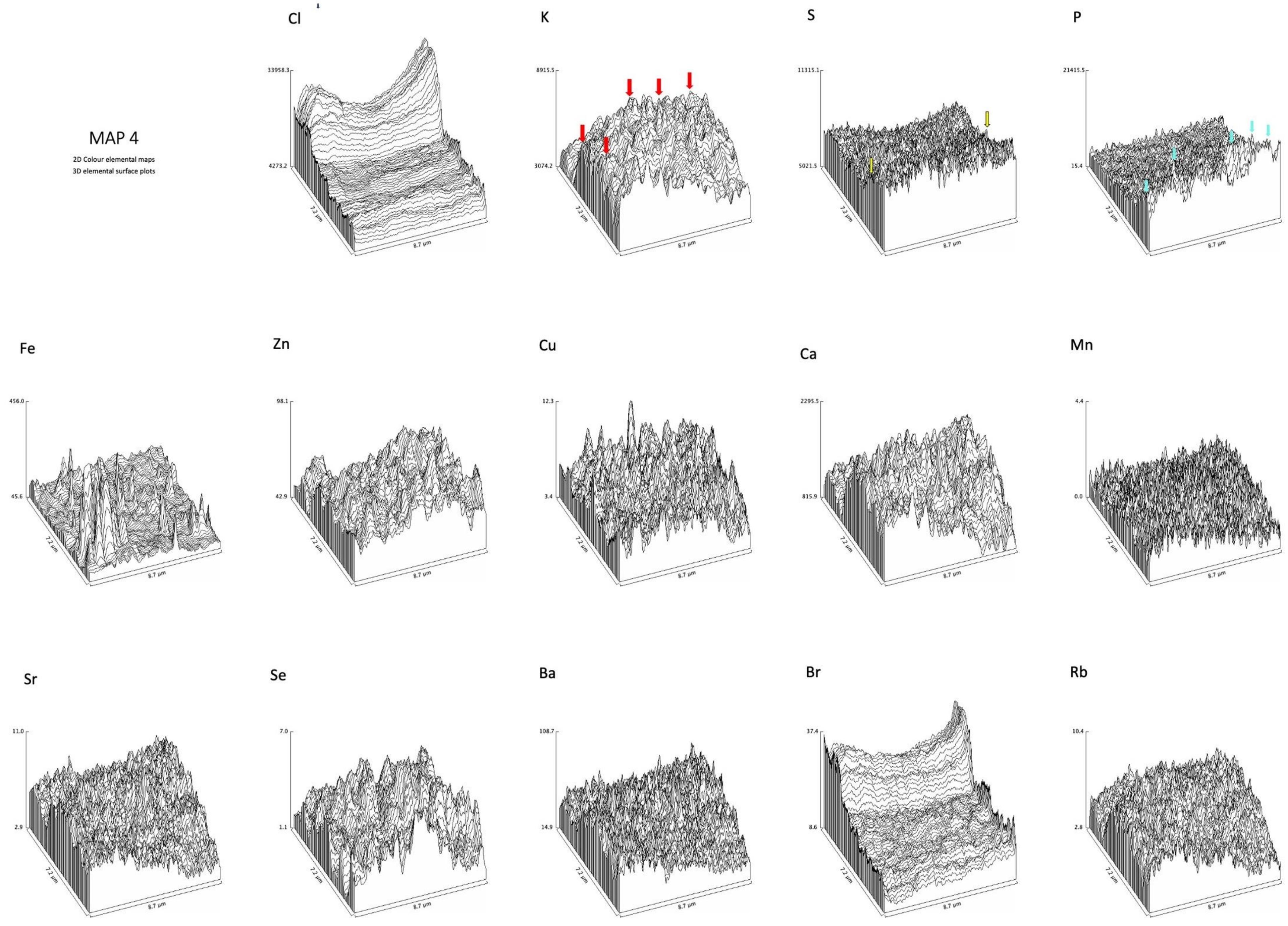
MAP 4
2D Colour elemental maps
3D elemental surface plots
Cl
33958.3
4273.2
K
8915.5
3074.2
S
11315.1
5021.5
P
21415.5
15.4
Fe
456.0
45.6
Zn
98.1
42.9
Cu
12.3
3.4
Ca
2295.5
815.9
Mn
4.4
0.0
Sr
11.0
2.9
Se
7.0
1.1
Ba
108.7
14.9
Br
37.4
8.6
Rb
10.4
2.8
7.2 µm
8.7 µm

MAP 5

2D Colour elemental maps
3D elemental surface plots

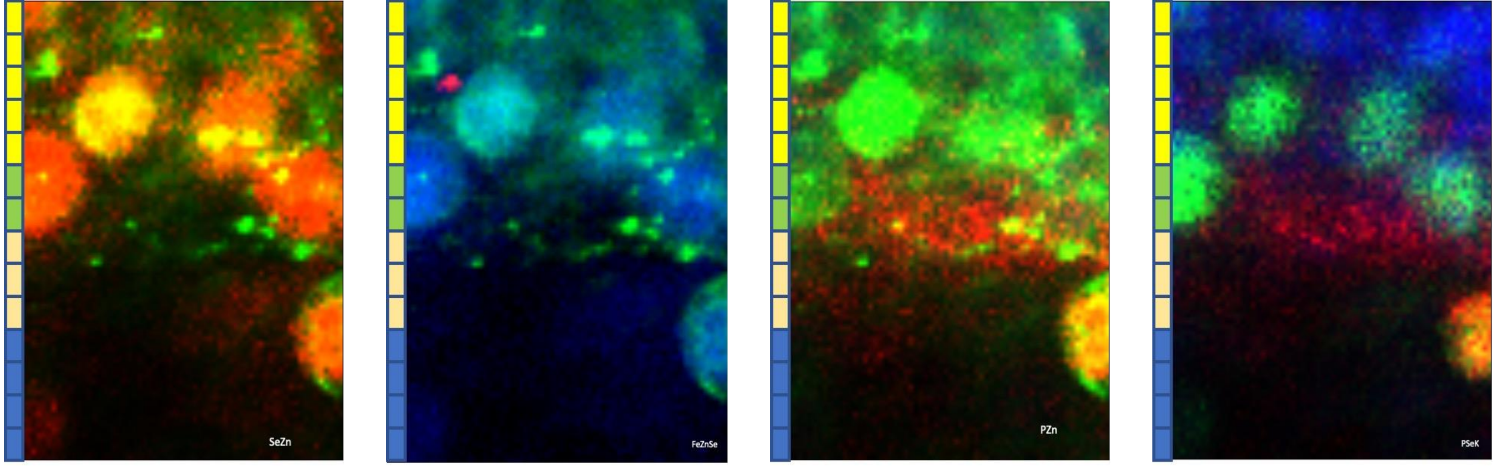

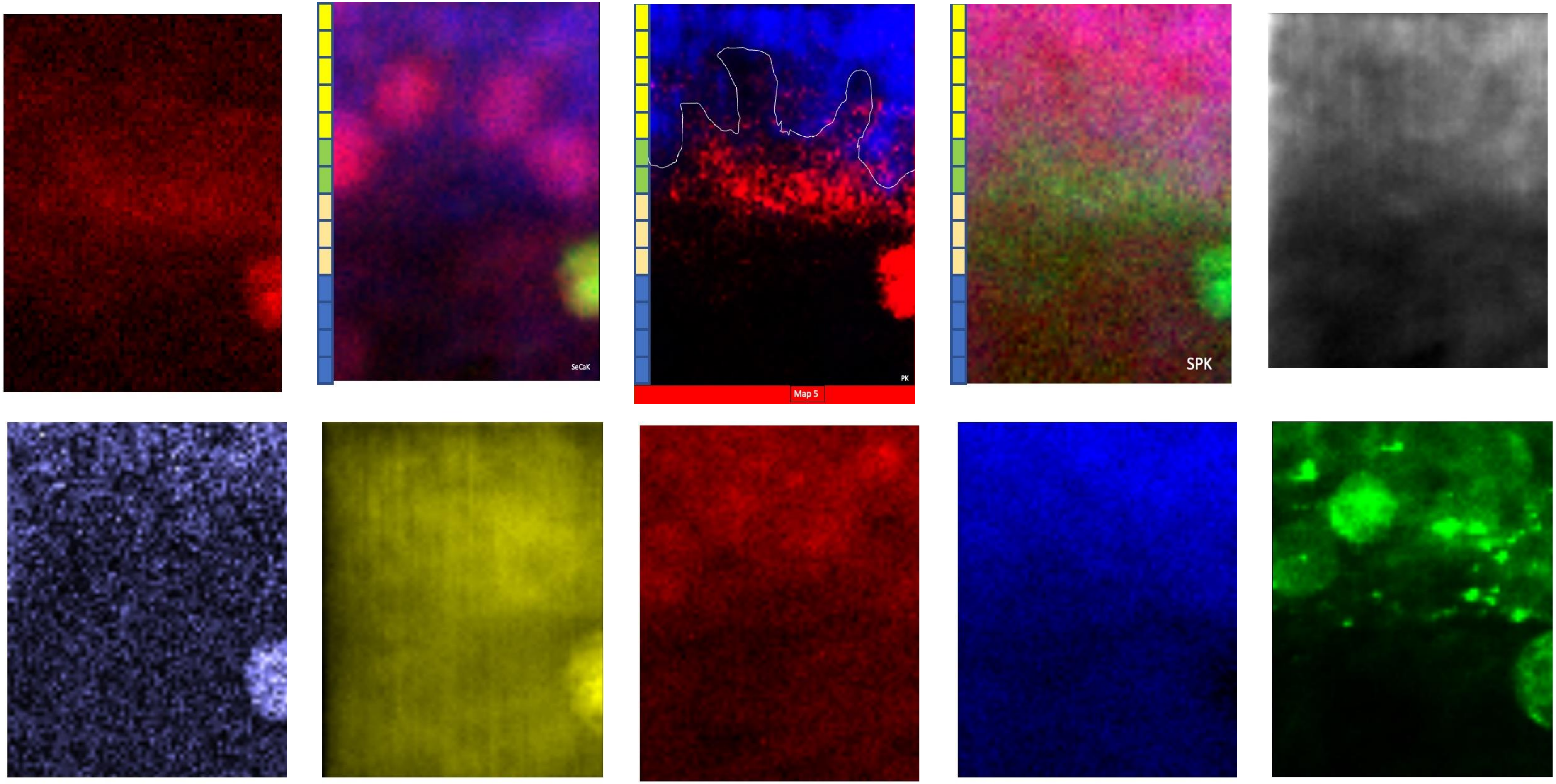

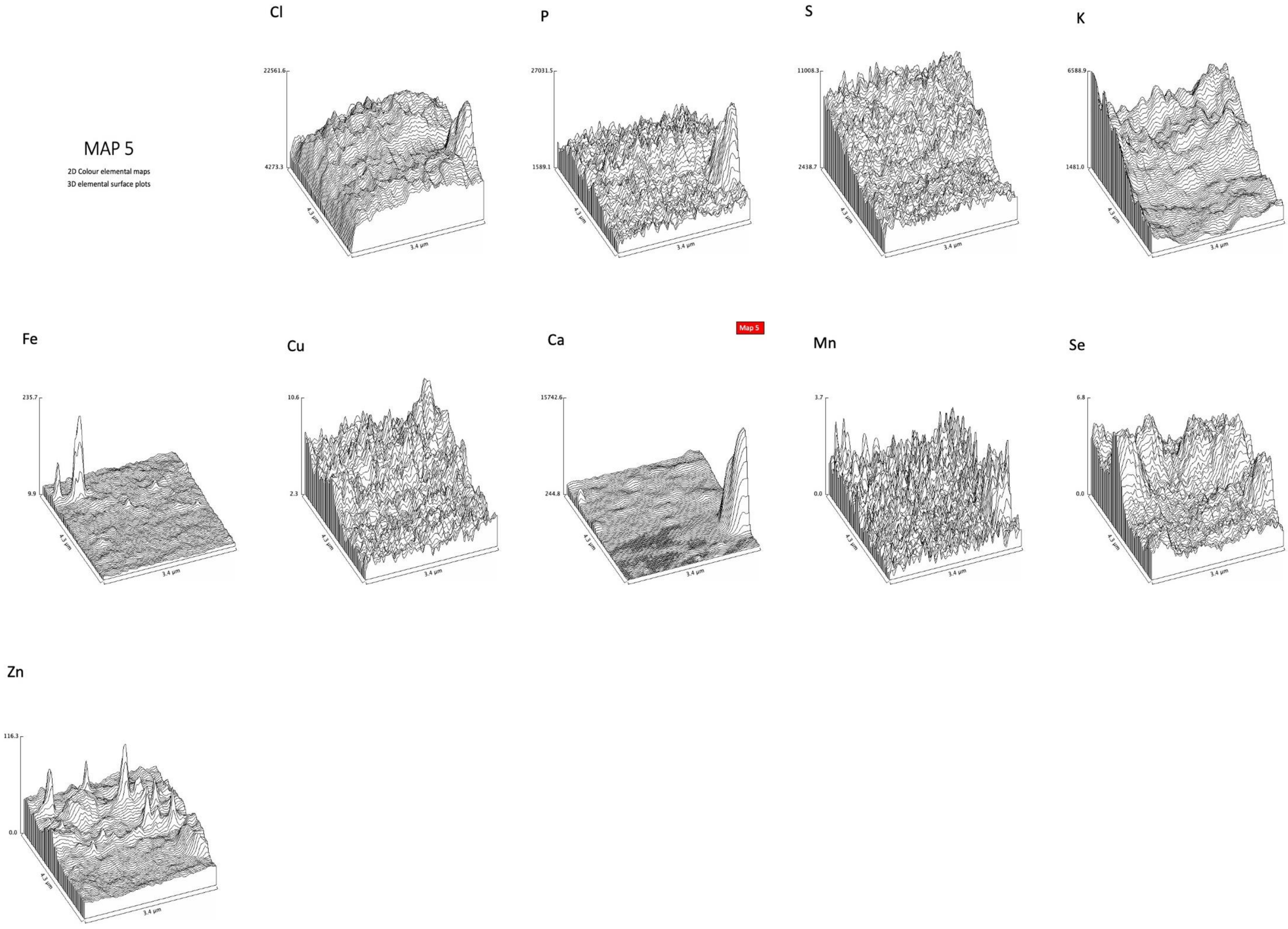
MAP 5
2D Colour elemental maps
3D elemental surface plots
Cl
22561.6
4273.3
4.3 µm
3.4 µm
P
27031.5
1589.1
4.3 µm
3.4 µm
S
11008.3
2438.7
4.3 µm
3.4 µm
K
6588.9
1481.0
4.3 µm
3.4 µm
Fe
235.7
9.9
4.3 µm
3.4 µm
Cu
10.6
2.3
4.3 µm
3.4 µm
Ca
15742.6
244.8
4.3 µm
3.4 µm
Map 5
Mn
3.7
0.0
4.3 µm
3.4 µm
Se
6.8
0.0
4.3 µm
3.4 µm
Zn
116.3
0.0
4.3 µm
3.4 µm

MAP 6

2D Colour elemental map
3D elemental surface plots

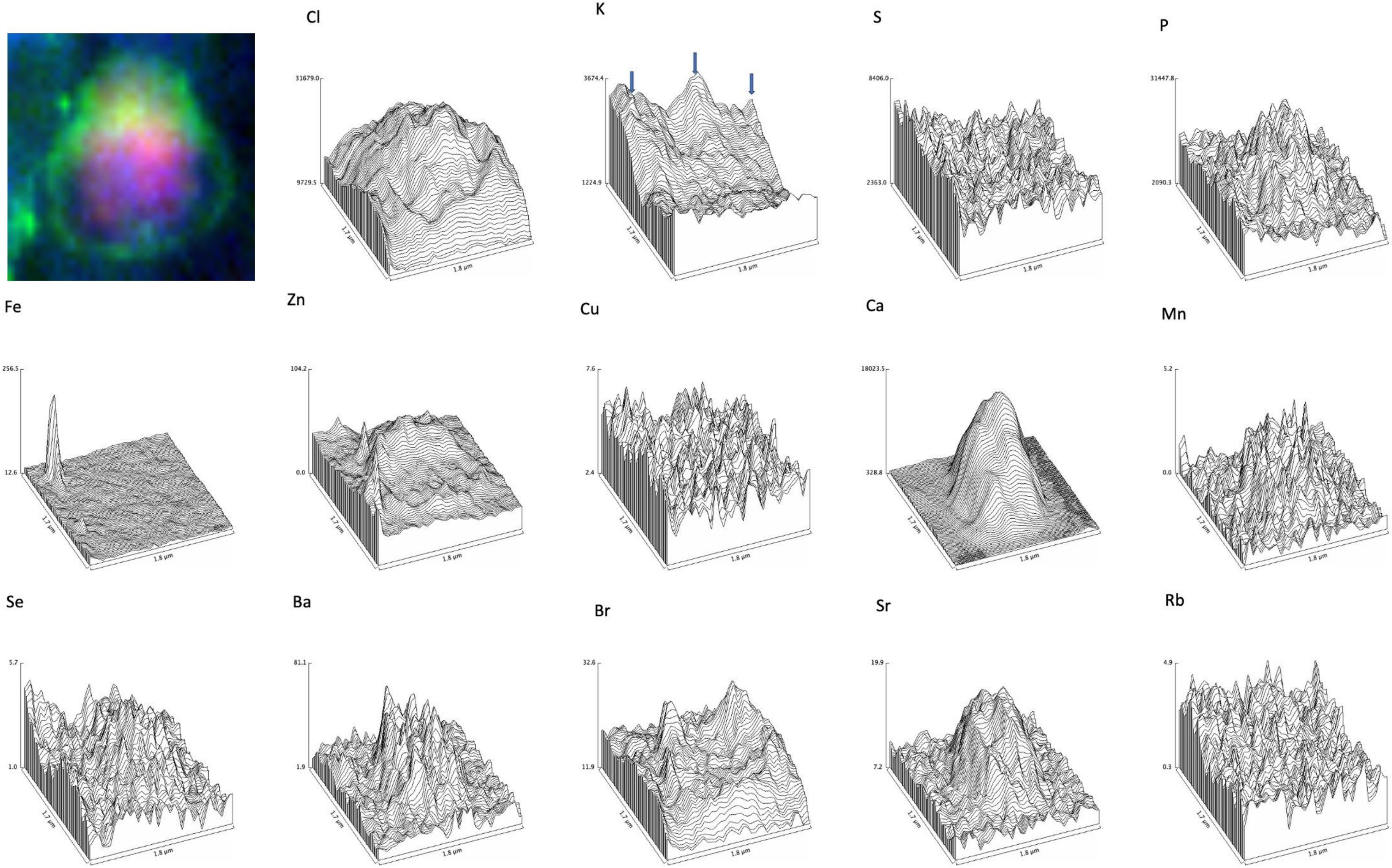

Supplement: Supplementary file 1 — Supplementary Material 1 [file 41598_2025_11678_MOESM1_ESM.pdf]
